# The Japanese Clinical Practice Guideline for acute kidney injury 2016

**DOI:** 10.1186/s40560-018-0308-6

**Published:** 2018-08-13

**Authors:** Kent Doi, Osamu Nishida, Takashi Shigematsu, Tomohito Sadahiro, Noritomo Itami, Kunitoshi Iseki, Yukio Yuzawa, Hirokazu Okada, Daisuke Koya, Hideyasu Kiyomoto, Yugo Shibagaki, Kenichi Matsuda, Akihiko Kato, Terumasa Hayashi, Tomonari Ogawa, Tatsuo Tsukamoto, Eisei Noiri, Shigeo Negi, Koichi Kamei, Hirotsugu Kitayama, Naoki Kashihara, Toshiki Moriyama, Yoshio Terada, The Japanese Clinical Practice Guideline for Acute Kidney Injury 2016 Committee, Kiyoshi Mori, Yoshihiro Taniyama, Shu Wakino, Hideo Yasuda, Shinji Kume, Tadashi Sofue, Kiichiro Fujisaki, Hideaki Shima, Koji Tomori, Taro Horino, Yusuke Watanabe, Hiroki Hayashi, Takeshi Moriguchi, Kazuto Yamashita, Ryota Inokuchi, Kensuke Nakamura, Yoshitaka Hara, Kengo Furuichi, Sho Sasaki, Takayuki Tsuji, Hiroyuki Yamada, Sayoko Yonemoto, Taka-aki Nakada, Noriyuki Hattori, Tetsushi Yamashita, Zentaro Kiuchi, Mariko Sawada, Masaki Takahashi, Masanori Tani, Yusuke Nakazawa, Masaki Nunoyama, Tsuguya Fukui, Seiichi Matsuo, Shoichi Maruyama, Motoko Yanagita, Kazuhiko Tsuruya

**Affiliations:** 1 Department of Acute Medicine, The University of Tokyo, Tokyo, Japan; 2 Department of Anesthesiology and Critical Care Medicine, Fujita Health University School of Medicine, Toyoake, Aichi Japan; 3 Department of Nephrology, Wakayama Medical University, Wakayama, Japan; 4 Department of Emergency and Critical Care Medicine, Tokyo Women’s Medical University Yachiyo Medical Center, Chiba, Japan; 5 Kidney Center, Department of Surgery, Nikko Memorial Hospital, Hokkaido, Japan; 6 Clinical Research Support Center, Tomishiro Central Hospital, Okinawa, Japan; 7 Department of Nephrology, Fujita Health University School of Medicine, Toyoake, Aichi Japan; 8 Department of Nephrology and General Internal Medicine, Saitama Medical University, Saitama, Japan; 9 Division of Anticipatory Molecular Food Science and Technology, Department of Diabetology and Endocrinology, Kanazawa Medical University, Kanawaza, Ishikawa Japan; 10 Department of Community Medical Supports, Tohoku Medical Megabank Organization, Tohoku University, Sendai, Japan; 11 Division of Nephrology and Hypertension, St. Marianna University School of Medicine, Kawasaki, Kanagawa Japan; 12 Department of Emergency and Critical Care Medicine, University of Yamanashi School of Medicine, Yamanashi, Japan; 13 Blood Purification Unit, Hamamatsu University Hospital, Hamamatsu, Japan; 14 Department of Kidney Disease and Hypertension, Osaka General Medical Center, Osaka, Japan; 15 Nephrology and Blood Purification, Saitama Medical Center, Saitama Medical University, Saitama, Japan; 16 Department of Nephrology, Graduate School of Medicine, Kyoto University, Kyoto, Japan; 17 Department of Nephrology and Endocrinology, The University of Tokyo, Tokyo, Japan; 18 Division of Nephrology and Rheumatology, National Center for Child Health and Development, Tokyo, Japan; 19 Department of Nephrology, Shizuoka Children’s Hospital, Shizuoka, Japan; 20 Department of Nephrology and Hypertension, Kawasaki Medical School, Okayama, Japan; 21 Health Care Division, Health and Counseling Center, Osaka University, Osaka, Japan; 22 Department of Endocrinology, Metabolism and Nephrology, Kochi Medical School, Kochi University, Kohasu, Oko-cho, Nankoku, 783-8505 Japan

**Keywords:** Acute kidney injury, Atrial natriuretic peptide, Biomarker, Blood purification, Long-term follow-up, Nafamostat mesilate

## Abstract

Acute kidney injury (AKI) is a syndrome which has a broad range of etiologic factors depending on different clinical settings. Because AKI has significant impacts on prognosis in any clinical settings, early detection and intervention are necessary to improve the outcomes of AKI patients. This clinical guideline for AKI was developed by a multidisciplinary approach with nephrology, intensive care medicine, blood purification, and pediatrics. Of note, clinical practice for AKI management which was widely performed in Japan was also evaluated with comprehensive literature search.

## CQ1: What is the concept of AKI, and what are the key elements of its clinical practice?

*Recommendation*: AKI is a syndrome associated with a broad spectrum of diseases and a variety of underlying pathologies. Therefore, differentiation of the causes and elimination of the reversible factors are always required.

*Strength of recommendation*: Not graded

*Quality of evidence*: D

### Commentary

In the past, the pathology associated with sudden renal impairment was characterized as an acute renal failure (ARF). However, in the 2000s, the joint efforts of specialists in fields including nephrology, intensive care medicine, and cardiovascular medicine led to the introduction of a novel concept called acute kidney injury (AKI). Although both ARF and AKI designate clinical conditions that present with sudden renal impairment and renal tissue damage, their respective clinical backgrounds leading to onset are thought to differ. In cases of ARF, high invasiveness is assumed to lead to sudden renal impairment in patients with relatively few comorbidities. In addition, as ARF is thought to be essentially a reversible disease, there was little awareness of its poor outcomes; greater attention was paid to the differentiation of the causes and to the countermeasures against the complications associated with renal failure than to the need for early detection. However, as medical care progressed, patients such as high-risk elderly subjects who were not deemed to be candidates for invasive therapy came to be treated in intensive care units (ICUs). Eventually, there grew to be widespread awareness of the increase in cases of sudden kidney injury comorbid with sepsis and multiple organ failure and of the incredibly poor outcomes in these cases. This led to kidney injury as a subset of multiple organ failure to be reconsidered as AKI in intensive care medicine. Thus, AKI was proposed as a novel disease concept to emphasize early diagnosis and early intervention for the improvement of prognoses.

Meanwhile, the RIFLE [1], AKIN [2], and KDIGO [3] diagnostic criteria were introduced in an effort to establish unified international diagnostic criteria. The present guideline recommends the use of the KDIGO diagnostic criteria (see the “CQ2-1: Should the diagnosis of AKI be based on the KDIGO diagnostic criteria?” section). However, these criteria are based solely on the serum creatinine (sCr) and urine output; they do not take into account the cause or site of the kidney injury or the location and mode of onset of AKI. Thus, as AKI refers to a syndrome with a broad spectrum of diseases and a variety of pathophysiologies, it calls for constant differentiation of the causes and elimination of the reversible factors. The KDIGO Clinical Practice Guideline for AKI [3] also recommends searching for and assessing the cause of the syndrome whenever possible, particularly in regard to reversible causes (recommendations 2.1.3 and 2.3.1).

## CQ2-1: Should the diagnosis of AKI be based on the KDIGO diagnostic criteria?

*Recommendation*: The KDIGO criteria are superior to the RIFLE criteria and to the AKIN criteria in predicting survival outcomes; therefore, we suggest using the KDIGO criteria to diagnose AKI. However, it is unknown which criteria should be used to predict the renal outcomes.

*Strength of recommendation*: 2

*Quality of evidence*: C

### Summary of evidence

We identified 11 observational studies that compared the KDIGO with the AKIN and RIFLE criteria and that assessed death as an outcome. However, they did not assess the initiation of dialysis. In these 11 observational studies, the comparisons of the AKI diagnosis based on the KDIGO criteria versus those based on the RIFLE and AKIN criteria showed that the KDIGO criteria are more precise than, or as precise as, the RIFLE and AKIN criteria in reflecting the in-hospital mortality.

### Commentary

In the past, acute renal failure (ARF) was diagnosed and classified according to several different criteria. In response to the growing call for unified international diagnostic criteria, the Acute Dialysis Quality Initiative (ADQI) published the RIFLE (Risk, Injury, Failure, Loss, End-stage kidney disease) criteria in 2004 (Table 1) [1, 4]. The RIFLE criteria distinguished three degrees of severity (risk, injury, and failure), with the latter defined as an increase in the serum creatinine (sCr), a decline in the glomerular filtration rate (GFR), and a reduction in the urine output, and two types of clinical outcomes (loss and end-stage kidney disease). In 2004, the members of the International Society of Nephrology, the American Society of Nephrology, the National Kidney Foundation (in the USA), and the European Society of Intensive Care Medicine founded the Acute Kidney Injury Network (AKIN); as a replacement for the term ARF, the AKIN advocated the concept of acute kidney injury (AKI), which encompasses earlier stages of kidney injury. On the other hand, after the RIFLE criteria were published, a mere 0.3 mg/dL increase in sCr was reported to affect the survival prognosis and the clinical course of AKI [5, 6].

**Table 1:** RIFLE criteria

	GFR criteria	Urine output criteria
Risk	Increase in sCr ≥ 1.5 × baseline or decrease in GFR ≥ 25%	UO < 0.5 mL/kg/h × 6 h
Injury	Increase in sCr ≥ 2.0 × baseline or decrease in GFR ≥ 50%	UO < 0.5 mL/kg/h × 12 h
Failure	Increase in sCr ≥ 3.0 × baseline or an absolute sCr ≥ 4.0 mg/dL with an acute rise of at least 0.5 mg/dL or decrease in GFR ≥ 75%	UO < 0.3 mL/kg/h × 24 h or anuria × 12 h
Loss	Complete loss of kidney function > 4 weeks	
ESKD	End-stage renal disease (dialysis dependent > 3 months)	

*GFR* glomerular filtration rate, *sCr* serum creatinine, *ESKD* end-stage kidney disease, *UO* urine output

In 2007, the AKIN proposed the AKIN criteria, which were a revision of the RIFLE criteria (Table 2) [2]. The AKIN criteria included milder increases in sCr (0.3 mg/dL) and added the time course of the sCr increase (within 48 h) to the diagnostic criteria. By contrast, a reduced GFR was removed from the RIFLE criteria. In addition, while both the AKIN criteria and the RIFLE criteria included the urine output, the AKIN criteria specified that when making a diagnosis based on the urine output alone, urinary tract obstructions and easily reversible causes of a reduced urine output were to be excluded and an adjustment was to be made for the body fluid volume. In addition, the RIFLE criteria’s loss and end-stage kidney disease were judged to be the outcomes of AKI and were removed from the AKIN criteria’s staging system. Furthermore, patients who had started renal replacement therapy (RRT) became classified as stage 3 regardless of their sCr and urine output prior to the RRT initiation.

**Table 2:** AKIN criteria

Definition	1. Increased in SCr of ≥ 0.3 mg/dL (48 h)	
	2. sCr changes ≥ 1.5 × baseline (48 h)	
	3. UO < 0.5 mL/kg/h × 6 h	
	sCr criteria	UO criteria
Stage 1	Increased in sCr of ≥ 0.3 mg/dL or increase to 1.5–2.0 × baseline	UO < 0.5 mL/kg/h × 6 h
Stage 2	Increase in sCr to 2.0–3.0 × baseline	UO < 0.5 mL/kg/h × 12 h
Stage 3	Increase in sCr > 3.0 × baseline or sCr ≥ 4.0 mg/dL with an acute rise of at least 0.5 mg/dL or Initiation of RRT	UO < 0.3 mL/kg/h × 24 h or anuria × 12 h

*sCr* serum creatinine, *UO* urine output, *RRT* renal replacement therapy

In 2012, the Kidney Disease: Improving Global Outcomes (KDIGO) group assembled all the available evidence into their own clinical practice guideline for AKI and proposed the KDIGO criteria, which integrate the RIFLE and AKIN criteria (Table 3) [3]. The KDIGO criteria diverge from the AKIN criteria in that the time course for a 1.5-fold increase in sCr from baseline was changed from within 48 h to within 7 days. Thus, as the KDIGO criteria encompass more gradual increases in sCr, they have made the number of patients diagnosed with AKI likely to increase.

**Table 3:** KDIGO criteria

Definition	1. Increased in SCr of ≥ 0.3 mg/dL (48 h) 2. sCr changes ≥ 1.5 × baseline (7 days) 3. UO < 0.5 mL/kg/h × 6 h	
	sCr criteria	UO criteria
Stage 1	Increased in sCr of ≥ 0.3 mg/dL or increase to 1.5–1.9 × baseline	UO < 0.5 mL/kg/h × 6 h
Stage 2	Increase in sCr to 2.0–2.9 × baseline	UO < 0.5 mL/kg/h × 12 h
Stage 3	Increase in sCr > 3.0 × baseline or sCr ≥ 4.0 mg/dL or Initiation of RRT	UO < 0.3 mL/kg/h × 24 h or anuria × 12 h

*sCr* serum creatinine, *UO* urine output, *RRT* renal replacement therapy

As described above, three sets of diagnostic criteria for AKI have been proposed: the RIFLE, AKIN, and KDIGO criteria. The utility of the KDIGO criteria, the most recent of the three sets, has been compared with that of the two older sets. In a prospective, multicenter observational study of 3107 intensive care unit (ICU) patients, Luo et al. reported the percentages of patients diagnosed with AKI according to the RIFLE, AKIN, and KDIGO criteria using both the sCr and urine output and compared their in-hospital mortality rates [7]. The percentages of patients diagnosed with AKI according to the RIFLE, AKIN, and KDIGO criteria were 46.9, 38.4, and 51.0%, respectively; thus, the number of patients diagnosed with AKI was significantly higher when using the KDIGO criteria than when using either the AKIN or RIFLE criteria. The patients diagnosed with AKI based on the KDIGO criteria had poorer survival outcomes than those diagnosed using the AKIN criteria, although there was no significant difference in the survival outcomes of patients diagnosed using the RIFLE criteria. In a retrospective multicenter study of 1005 adult patients hospitalized for acute heart failure, Li et al. compared the percentages of patients diagnosed with AKI within 7 days of hospitalization using the KDIGO, AKIN, and RIFLE criteria, as well as the patients’ in-hospital mortality rates [8]. Using only the sCr criterion, the percentages of patients diagnosed with AKI according to the KDIGO, AKIN, and RIFLE criteria were 38.9, 34.7, and 32.1%, respectively. A total of 110 patients (10.9%) were diagnosed with AKI with the KDIGO criteria but not with the RIFLE or AKIN criteria. A total of 18.4% of the patients who died in the hospital were diagnosed with AKI according to the KDIGO criteria only; this group was at a high risk of in-hospital death. In a study of 1050 patients hospitalized for acute myocardial infarction, Rodrigues et al. compared the percentages of patients diagnosed with AKI according to the RIFLE and KDIGO criteria using the sCr criterion only, as well as their mortality rates [9]. A total of 14.8% of patients were diagnosed with AKI with the RIFLE criteria versus 36.6% with the KDIGO criteria. In comparison with patients without AKI, the 30-day and 1-year mortality hazard ratios for patients diagnosed with AKI according to the KDIGO criteria but not to the RIFLE criteria were 2.55 and 2.28, respectively.

In other studies of the AKI diagnostic criteria in hospitalized patients [10, 11], ICU patients [12–14], acute decompensated heart failure [15], patients after cardiac surgery [16], and sepsis [17], the KDIGO criteria were reported to be equal or superior to the RIFLE and AKIN criteria in their ability to predict the survival outcomes. Based on the above, the KDIGO criteria are considered to be more useful in their survival outcome prediction ability than the RIFLE or AKIN criteria for the diagnosis of AKI.

### Literature review

PubMed was searched for relevant studies published between January 1990 and July 2015, and papers related to the present CQ were identified from the search results.

## CQ2-2: When diagnosing AKI, how should an unknown baseline renal function be estimated?

*Recommendation*: Whenever possible, the baseline renal function should be determined using multiple methods, and the potential presence of chronic kidney disease (CKD) and other comorbidities should be assessed.

*Strength of recommendation*: 2

*Quality of evidence*: C

### Summary of evidence

Several methods have been suggested to estimate the baseline renal function. However, compared to the use of the known baseline function, all of these methods have been reported to yield a certain rate of false positives or false negatives in their AKI diagnoses and mortality predictions.

### Commentary

The diagnosis of acute kidney injury (AKI) requires the baseline renal function; however, in actual clinical practice, the patient’s history of examination and his/her baseline renal function are often unknown. These cases require an estimation of the baseline renal function, and many methods have been proposed (Table 4).

**Table 4:** Estimation of unknown baseline serum creatinine

Estimating baseline sCr method	Characteristics in the diagnosis of AKI	Reference
An estimated sCr determined by back-calculation using MDRD assuming a GFR of 75 mL/min/1.73 m^2^	Low specificity especially in CKD patients	[18–24]
An estimated sCr determined by back-calculation using MDRD assuming a GFR of 100 mL/min/1.73 m^2^	Very high sensitivity and very low specificity	[23]
The first admission sCr	Low sensitivity	[22]
A minimum inpatient sCr during the first 7 days	Low specificity	[22]
A minimum sCr during the first 7 days in the ICU	Low specificity although tendency to underestimate the AKI stage	[23]
An estimated sCr using multiple imputation methods such as sex, race, comorbidity (CKD, etc.), and a minimum inpatient sCr	High specificity	[19]
A minimum inpatient sCr		[11]
sCr = 1.0 mg/dL (male)/0.8 mg/dL (female)		[25]

*sCr* serum creatinine, *GFR* glomerular filtration rate, *CKD* chronic kidney disease

To compare the estimated baseline renal function with the known baseline renal function, we identified seven observational studies that used the AKI diagnosis as an outcome [18–24] and two observational studies that used the all-cause mortality as an outcome [20, 22]. While two of the seven studies included all hospitalized patients [19, 22], two of them limited the subjects to intensive care unit (ICU) patients [23, 24], two used only patients undergoing cardiac surgery [20, 21], and one study only included patients with cirrhosis [18]. All these studies assumed the lower limit of the normal renal function to be an estimated glomerular filtration rate (eGFR) of 75 mL/min/1.73 m^2^ (as suggested by the KDIGO Clinical Practice Guideline [3]) and examined a way to back-calculate the serum creatinine (sCr) based on the MDRD equation. In the six studies whose subjects included all hospitalized patients, the ICU patients only, and the cardiac surgery patients only, an assumed baseline renal function of eGFR 75 mL/min/1.73 m^2^ yielded false-positive AKI diagnoses. Four of these studies [19, 21, 23, 24] stated that false positives were especially frequent in patients with a known eGFR < 60 mL/min/1.73 m^2^. On the contrary, in the study whose subjects included cirrhosis patients only, an assumed baseline renal function of eGFR 75 mL/min/1.73 m^2^ yielded false-negative AKI diagnoses. In a study that was not taken into account due to its unsuitable outcome, Zavada et al. indicated that the estimated sCr was higher than the known sCr in young people [25]. The two observational studies that used the all-cause mortality as an outcome reported that the mortality rates were reduced by sCr estimation methods that frequently yielded false-positive AKI diagnoses, while the mortality rates were increased by estimation methods that frequently yielded false-negative diagnoses.

In conclusion, there is currently no specific baseline sCr estimation method on par with a measured baseline sCr. The easy method that involves the calculation of the sCr based on an eGFR of 75 mL/min/1.73 m^2^ is tolerable; however, this method often overestimates the sCr in young people and cirrhosis patients and underestimates it in chronic kidney disease (CKD) patients. Therefore, we suggest that whenever possible, the baseline renal function should be determined using multiple methods while also confirming whether the CKD and other comorbidities are present based on methods such as image searches to check for renal atrophy.

### Literature review

PubMed was searched for relevant studies published up to July 2015, and papers related to the present CQ were identified from the search results.

## CQ2-3: Should the AKI staging with the urine output be included in addition to the serum creatinine for the predictions of the AKI outcomes?

*Recommendation*: In the RIFLE, AKIN, and KDIGO criteria, the inclusion of the urine output along with the serum creatinine to determine the AKI stage yields more accurate reflections of the survival outcomes and the renal outcomes than the determination of the AKI stage based on the serum creatinine alone. Therefore, we suggest that the AKI staging should involve the urine output whenever possible.

*Strength of recommendation*: 2

*Quality of evidence*: B

### Summary of evidence

We identified seven observational studies that used death as an outcome. In the studies of ICU patients, the inclusion of the urine output as a criterion significantly improved the survival outcome predictions. In one of these studies, the renal outcome prediction was also improved; however, a study of patients after cardiac surgery indicated a potential for overdiagnosis. Because no study of outpatients or general ward patients has been conducted, it is unclear whether the above results can be generalized.

### Commentary

During the 10 years since the concept of AKI was introduced, three sets of diagnostic criteria/classifications have been proposed: the RIFLE, AKIN, and KDIGO. All these criteria sets enable the diagnosis and staging of AKI based on changes in the serum creatinine (sCr) or the urine output [1–3]. In many previous clinical studies, AKI was diagnosed and staged according to the sCr alone, and a slight increase in the sCr was reported to affect the survival outcomes [3, 6]. However, few clinical studies have used the urine output as a criterion for the diagnosis and staging of AKI. Therefore, we examined whether the urine output reflects the survival outcomes of AKI as accurately as the sCr and whether the inclusion of the urine output in the determination of the AKI stage reflects the survival outcomes more accurately than a determination based on the sCr alone.

To compare the sCr and the urine output, we adopted seven observational studies that used death as an outcome [26–32]. All these studies were conducted in intensive care units (ICUs); none of them involved outpatients or patients in general wards. Regarding the AKI diagnostic criteria, three studies used the RIFLE criteria [26, 27, 30], two used the AKIN criteria [31, 32], and two used the KDIGO criteria [28, 29]. In six of these studies, the inclusion of the urine output with the sCr in the AKI diagnosis significantly improved the survival outcome predictions [27–32]; furthermore, in one of these six studies, the renal outcome prediction ability was also improved [27].

In an analysis of 155,624 patients hospitalized in ICUs on an emergency admission, Harris et al. reported that the urine output was a more powerful predictor of the survival outcomes than the sCr [26]. In a study of 32,045 adult ICU patients classified according to the KDIGO sCr and urine output criteria, Kellum et al. demonstrated that patients who fulfilled both the sCr and urine output criteria were at the highest risk of death and the initiation of permanent renal replacement therapy (RRT), while isolated oliguria was associated with a long-term risk of death even when the sCr criterion was not fulfilled [27]. Similarly, in an analysis of 390 septic shock patients, Leedahl et al. reported that persistent oliguria was a risk factor for death by day 28 [28]. In a study of 260 ICU patients, Wlodzimirow et al. compared the combined use of the RIFLE’s sCr and urine output criteria (RIFLEsCr+urine output) with the use of the sCr criterion alone (RIFLEsCr); they reported that the RIFLEsCr was associated with a delayed AKI diagnosis and higher mortality [29]. Furthermore, Han et al. [30] and Macedo et al. [31] also reported that the addition of the urine output criterion enabled a more accurate AKI diagnosis than the use of the sCr criterion alone. Although some studies have featured different urine output criterion values, overall, the assessment of the urine output has been shown to improve the accuracy of the AKI diagnosis. However, in a comparison of the sCr criterion alone with the urine output criterion alone for the diagnosis of AKI in patients after cardiac surgery, Lagny et al. indicated that the use of the urine output criterion alone could lead to overdiagnosis [32]. In a recent multicenter prospective study that assessed the association between the hourly urine output and mortality, Vaara et al. reported that patients who fulfilled both the sCr and urine output criteria had the highest rate of RRT initiation and the highest 90-day mortality, while isolated oliguria was associated with poor outcomes; these results affirm the importance of measuring the hourly urine output and the need to combine the urine output criterion with the sCr criterion [33]. Moreover, in using the urine output criterion in the diagnosis and staging of AKI, there is a concern that the use of diuretics may change the urine output, causing underestimation of the AKI severity. However, in their analysis of the effects of diuretics on the AKI diagnosis, Han et al. reported that the inclusion of the urine output criterion alongside the sCr criterion played an additional role in the diagnosis and staging of AKI regardless of whether diuretics were used [30].

To summarize the above studies, the inclusion of the urine output along with the sCr to determine the AKI stage improves the sensitivity of the AKI diagnosis and yields more accurate reflections of the survival and renal outcomes than AKI staging based on the sCr alone. Therefore, we suggest that AKI staging should involve the urine output whenever possible.

### Literature review

PubMed was searched for relevant studies published between January 1990 and August 2015, and papers related to the present CQ were identified from the search results.

## CQ3-1: What should be assessed as risk factors for AKI development in cardiac surgery?

*Recommendation*: We suggest that factors such as age, preoperative renal dysfunction, and the duration of the cardiopulmonary bypass should be assessed as risk factors.

*Strength of recommendation*: 2

*Quality of evidence*: C

### Summary of evidence

We identified seven papers that assessed the risk of development of AKI in cardiac surgery. All of them were observational studies. Certain observational studies have stated that transcatheter aortic valve replacement (TAVR) and transcatheter aortic valve implantation (TAVI), which have become more common with the recent aging of society, do not match the same risk of AKI observed in cardiac surgery.

### Commentary

#### Background

Acute kidney injury (AKI) is a comorbidity that complicates the perioperative management of body fluid; the risk of AKI development is reported to be particularly high in cardiac surgery [34]. In Hu et al.’s meta-analysis of 91 studies of cardiac surgery, the incidence of postoperative AKI was 22.3%, while 2.3% of the patients required renal replacement therapy (RRT). Furthermore, the in-hospital mortality of patients who developed AKI following cardiac surgery was 10.7%, and the mortality in long-term observation (1–5 years) was 30.0% [35]. Therefore, assessment of the risk of AKI development is crucial for patients scheduled to undergo cardiac surgery. Nearly all the relevant studies have been observational studies, which make them insufficient to demonstrate strong evidence; nevertheless, they have identified several potential risk factors (Table 5).

**Table 5:** Risk factors for AKI development in cardiac surgery

Reference	Author, year	Aging	Obesity	Diabetes	Hypertension	Preoperative anemia	Preoperative renal impairment	Cardiopulmonary bypass duration
[37]	Kristovic et al. 2015	○	○	△	×	–	–	–
[38]	Joung et al. 2014	○	×	×	△	△	△	○
[39]	Ng et al. 2014	○	○	△	○	○	○	○
[36]	Ozkaynak et al. 2014	○	○	×	×	○	–	○
[43]	Kumar et al. 2012	–	–	–	–	–	–	○
[40]	Parolari et al. 2012	○	–	–	–	–	○	–
[41]	Huang et al. 2011	○	–	○	–	–	○	–

○ risk, △ risk without significance, × not risk, − not evaluated

#### Aging

The aging of patients who undergo cardiac surgery may make their perioperative management more difficult. In a prospective observational study, Ozkayanak et al. reported that the risk of development of AKI from cardiac surgery increased with age (odds ratio 1.022, 95% confidence interval 1.005–1.039) [36]. Nearly identical results have been demonstrated in retrospective observational studies [37, 38]. Regarding coronary artery bypass grafting (CABG), a prospective observational study limited to Asian patients showed that AKI developed significantly more frequently in patients aged ≥ 70 years (odds ratio 1.350, 95% confidence interval 1.085–1.679) [39]. A retrospective observational study of patients who had undergone cardiac surgery with a cardiopulmonary bypass also reported age as a significant risk factor for AKI development [40]. The risk of AKI development should be considered while treating elderly patients undergoing cardiac surgery.

#### Preoperative renal impairment

Preoperative renal dysfunction is known as a risk factor for perioperative AKI development. Observational studies of cardiac surgery patients have also reported that pre-AKI renal dysfunction was a potential risk factor for AKI development. Huang et al. reported that CKD stage G3 (odds ratio 1.68, 95% confidence interval 1.12–2.52) and CKD stage G4 (odds ratio 3.01, 95% confidence interval 1.57–6.03) were risk factors for AKI development after cardiac surgery [41]. In a prospective observational study of CABG patients, Guenancia et al. reported that a higher preoperative estimated glomerular filtration rate (eGFR) was associated with a lower risk of AKI development (odds ratio 0.97, 95% confidence interval 0.96–0.99) [42]. In another prospective observational study of CABG patients, Ng et al. reported that a higher preoperative serum creatinine (sCr) value was associated with an increased risk of AKI development (odds ratio 1.003, 95% confidence interval 1.001–1.006) [39].

#### Duration of cardiopulmonary bypass

A cardiopulmonary bypass (CPB) creates an extracorporeal environment and a non-physiological state in which a constant blood flow is maintained with a pump, independently from the heartbeat. In typical CPBs, the blood is diluted by 20–50% to reduce the hemoglobin concentration. During CPBs, the renal blood flow is affected by various factors, including hypothermia, blood dilution, hemolysis, microthrombi, and vasoactive drugs; these factors constrict the renal artery and reduce the renal blood flow. In a meta-analysis of nine studies on the correlation between the duration of CPBs in cardiac surgery and the development of AKI, the CPB duration was reported to be significantly associated with the development of AKI [43]. Off-pump surgery, which has become more common recently, could make the surgery less invasive for elderly heart disease patients in Japan. In a meta-analysis of randomized controlled trials (RCTs) involving CABG patients, Seabra et al. reported that compared to on-pump CABG, off-pump CABG significantly inhibited the postoperative AKI onset; however, no significant association was observed with the need for dialysis [44]. In an RCT that observed the long-term renal outcomes, the incidence of AKI development within 30 days after surgery was significantly lower after off-pump CABG (17.5% vs 20.8%, 95% confidence interval: 0.72–0.97); however, at 1 year, there was no difference in the percentages of patients with a reduced eGFR. Therefore, recent RCTs have failed to sufficiently prove the efficacy of off-pump CABG for renoprotection.

#### Other risk factors

In addition to the risk factors stated above, observational studies have also assessed obesity, diabetes, hypertension, and anemia as potential risk factors; however, due to contradictory results, no conclusions have been reached [38–42]. Recently, transcatheter aortic valve implantation (TAVI) and transcatheter aortic valve replacement (TAVR) have become more common, since they can be performed with minimal invasiveness in the elderly and high-risk patients. In a meta-analysis of 13 studies, Elhmidi et al. reported that preoperative renal impairment was a significant risk factor for post-TAVI AKI development [45].

### Literature review

PubMed was searched for relevant studies published between March 2011 and December 2015, and papers related to the present CQ were identified from the search results. The literature published before March 2011 was referenced from the KDIGO Clinical Practice Guideline for AKI.

## CQ3-2: What should be assessed as risk factors for AKI development in non-cardiac surgery?

*Recommendation*: In liver transplantation, we suggest that the preoperative model for end-stage liver disease (MELD) score, the intraoperative blood transfusion volume, the intraoperative hypotension, and the use of vasopressors should be assessed as risk factors for AKI development. The potential risk factors related to other non-cardiac surgeries are unknown.

*Liver transplantation*:

*Strength of recommendation*: 2

*Quality of evidence*: C

*Other surgeries*:

*Strength of recommendation*: Not graded

*Quality of evidence*: D

### Summary of evidence

Among ten observational studies on the development of AKI following liver transplantation, five studies demonstrated a significant association between the development of AKI and the intraoperative blood transfusion volume. Two studies excluded chronic kidney disease (CKD), while two others found CKD to be a significant risk factor for AKI development. Two studies demonstrated that the MELD score and intraoperative hypotension or the use of vasopressors were associated with the development of AKI. Only three studies about lung transplantation and AKI development were found, and these studies did not demonstrate a consistent trend.

### Commentary

#### Background

The development of AKI is significantly associated with increased mortality. This lends great clinical significance to the development of AKI following non-cardiac surgery as well as cardiac surgery. Therefore, it is crucial to determine the incidence rate of AKI, the risk factors for its development, and its association with prognoses. Despite the existence of several studies about the development of AKI after liver transplantation, nearly all have been observational studies. Furthermore, there have been few studies on the development of AKI after non-cardiac surgeries other than liver transplantation.

#### Liver transplantation

In liver disease, the development of AKI is generally a risk factor for the progression of hepatic dysfunction and increased mortality [46]. In liver transplantation, one of the most invasive liver surgery procedures, postoperative AKI is associated with mortality; therefore, it is crucial to assess the risk factors that predict its development. Many studies have reported the incidence of AKI after liver transplantation; however, it has ranged greatly, between 17 and 95% [47]. Recent investigations have primarily used the AKIN classification system; in retrospective studies published between 2013 and 2015, the incidence of the post-liver transplantation development of AKI ranged from 10 to 30% [48–53]. In 2014, Leithead et al. reported an investigation of the AKI onset among 1152 patients who had undergone liver transplantation [48]. The study defined AKI as the progression to KDIGO stage 2 or higher within 1 week after transplantation. Based on this definition, the incidence of AKI was 33.8%; factors such as the preoperative MELD score, preoperative hyponatremia, a preoperative BMI ≥ 30 kg/m^2^, intraoperative red blood cell transfusion, and a long warm ischemic time were identified as risk factors for AKI development [54]. In transplantation, the length of time from the stopping of the organ blood flow to the resumption of the blood flow following transplantation is defined as the ischemic time; the exposition of the organ to an ischemic state, particularly at a normal temperature, increases the likelihood that cells will die. This time is called the warm ischemic time; the ideal time is 0 min for the heart and liver and 30 min for the kidneys and lungs. In order to achieve these ideal times, the organs must be cooled at an early stage to reduce the cellular metabolism. The fact that these unique liver transplantation parameters are associated with AKI is fascinating in terms of organ crosstalks. There have been ten observational studies on the development of AKI following liver transplantation [48–53, 55–58]. Five of them have reported an intraoperative red blood cell transfusion as an independent risk factor for AKI development, while two studies have reported the preoperative MELD score, intraoperative hypertension, and the use of vasopressors as independent risk factors. A retrospective cohort study in 2015 reported the same results in relation to liver resection [59]. In that study, 78 of the 642 patients who had undergone liver resection developed AKI (as defined according to the AKIN classification) within 72 h. Preoperative renal impairment, preoperative hypertension, and intraoperative red blood cell transfusion were identified as risk factors for AKI development. However, this study is the only one to have examined the development of AKI after liver resection to date.

#### Lung surgery

There have been three studies on AKI following lung surgery, and all of them have been retrospective cohort studies [60–62]. In one of them, George et al. assessed the need for postoperative RRT in a multicenter study of 12,108 patients and found an AKI incidence of 5.5%; increasing age, the male gender, a black ethnicity, a decreased preoperative renal function, a high preoperative bilirubin level, a preoperative comorbid lung disease, bilateral lung surgery, the use of intraoperative or postoperative extracorporeal membrane oxygenation (ECMO), and the ischemic time were identified as risk factors for AKI development [61]. Xue et al. examined the development of AKI of AKIN stage 1 or higher within 1 week after lung transplantation in 88 patients and found an AKI incidence of 53.4%. The proposed risk factors included aging, preoperative hypertension, an intraoperative low mean blood pressure, the intraoperative use of vasopressors, the intraoperative use of aprotinin, the use of intraoperative or postoperative ECMO, and a comorbid postoperative infection [60]. In an investigation of the onset of AKI classified by the RIFLE criteria as “Risk” or more severe within 1 week after lung cancer surgery, Licker et al. reported an AKI incidence of 6.8%. A low preoperative forced expiratory volume in 1 s (FEV 1.0%), a high ASA score, and the duration of the anesthesia were identified as risk factors. The ASA score refers to the physical status assessment score advocated by the American Society of Anesthesiologists [62].

#### Bariatric surgery

Bariatric surgery has recently become a popular surgical intervention for severe obesity, mainly in the West. As obesity itself triggers renal impairment, multiple studies have examined the development of AKI following bariatric surgery [63–65]. In a cohort of 590 patients, Morgan and Ho reported that AKI of AKIN stage 1 or higher had developed in 103 patients, which represented an incidence of 17.5%; the male gender, preoperative hypertension, and a high preoperative APACHE II score were identified as risk factors [64]. In a report of the outcomes in 1227 patients who underwent bariatric surgery at the Mayo Clinic between 2004 and 2011, the incidence of AKI (defined as a serum creatinine [sCr] increase of 0.3 mg/dL within 72 h) was 5.8%; the preoperative BMI and diabetes were identified as risk factors for AKI development [65].

#### Colorectal surgery

Causey et al. examined the development of AKI following colorectal surgery in a cohort of 339 patients who underwent colorectal surgery between 2001 and 2009 [66]. The incidence of AKI (defined as a postoperative increase in sCr of ≥ 50% from baseline) was 11.8%; intraoperative red blood cell transfusion was identified as a risk factor.

### Literature review

PubMed was searched for relevant studies published between March 2011 and December 2015, and papers related to the present CQ were identified from the search results. The literature published before March 2011 was referenced from the KDIGO Clinical Practice Guideline for AKI.

## CQ3-3: What should be assessed as risk factors for AKI development in heart failure?

*Recommendation*: Factors such as aging, renal impairment, and cardiac dysfunction should be assessed as risk factors.

*Strength of recommendation*: 2

*Quality of evidence*: C

### Summary of evidence

Among the 11 identified observational studies that included the development of AKI as an outcome, 5 were multicenter studies involving more than 1000 subjects. In multivariate analyses, the following risk factors were found to be significantly associated with AKI: comorbid CKD (4 studies), aging (4 studies), diabetes (3 studies), and cardiac dysfunction (3 studies). Other factors found to be associated with AKI were the diuretic resistance, hypotension (defined as a systolic blood pressure < 90 mmHg), and elevated urinary neutrophil gelatinase-associated lipocalin (NGAL) (2 studies each).

### Commentary

#### Background

In cardiovascular medicine, AKI has been recognized as worsening renal function (WRF) in heart failure patients. The interaction between heart failure and kidney failure has recently been defined as cardiorenal syndromes (CRS), which are classified into five types [67, 68]. Among them, AKI associated with acute heart failure is classified as CRS type 1. Furthermore, AKI caused by acute heart failure is considered to exacerbate the heart failure, causing a vicious cycle and a poor survival prognosis for patients with CRS type 1 [69]. Therefore, it is clinically crucial to identify the incidence and risk factors of CRS type 1.

#### Incidence of AKI in acute heart failure

Studies have found an inconsistent incidence of AKI in acute heart failure due to differing definitions of AKI. A retrospective cohort study in 2010 by Amin et al. featured 2098 enrolled patients (the largest cohort to date). In this study, the incidence of AKI—defined as an increase in sCr of ≥ 0.3 mg/dL during hospitalization—was 18.7% [70]. However, in a retrospective cohort study (*n* = 1010) in 2013 by Wang et al. that defined AKI as stage 1 or higher according to the AKIN classification, the incidence of AKI was 32.2% [71], while in a prospective cohort study (*n* = 1005) in 2012 by Zhou et al. that defined AKI according to the RIFLE classification, the AKI incidence was 44.3% [72]. Subsequent studies by Soyler et al. [73] that defined AKI as an increase in sCr of ≥ 0.3 mg/dL within 48–72 h after hospital admission, and by Tung et al. [74] that examined AKIN stage ≥ 1 AKI patients hospitalized for ST-segment elevation myocardial infarction (STEMI), reported AKI incidences of 19.0 and 19.6%, respectively. In a study that examined the differences in the incidence of AKI based on the different definitions of AKI, Li et al. reported that the incidences of AKI in a cohort of patients hospitalized for acute heart failure (*n* = 1498) according to the RIFLE, AKIN, and KDIGO criteria were 32.1, 34.7, and 38.9%, respectively [8]. Based on the above studies, the incidence of AKI in heart failure is considered to range between 20 and 40%.

#### Risk factors for AKI development in acute heart failure

Observational studies have identified a number of risk factors for AKI following heart failure. A recent prospective observational study demonstrated a significant association between AKI and elevated levels (≥ 12 ng/mL) of the tubular dysfunction marker NGAL [75]. In 11 existing observational studies, comorbid CKD, aging, comorbid diabetes, and cardiac dysfunction were identified as independent risk factors for AKI. Diuretic resistance, hypotension (defined as a systolic blood pressure < 90 mmHg), and an elevated urinary NGAL were also shown to be associated with AKI in two studies each (Table 6). The degree of CKD considered to present a risk of AKI development is an eGFR < 60 mL/min/1.73 m^2^ [70, 72, 74, 76] or a sCr level ≥ 104 μmol/L (1.17 mg/dL) [71]. In terms of age, one study stated that the odds ratio for the development of AKI increased by 1.17 (95% confidence interval 1.08–1.28) with every 10 years increase in age [70], while other studies have reported ages of ≥ 70 years [71] and ≥ 80 years [76] as risk factors. One study defined the degree of cardiac dysfunction considered a risk factor for AKI development as a left ventricular ejection fraction (LVEF) < 40% [70], while others set it at LVEF < 45% or a NYHA class IV [71, 72]. The extent of diuretic resistance that is considered a risk factor for AKI development has been defined as persistent pulmonary congestion despite repeated doses of 80 mg furosemide, the continuous administration of 240 mg of furosemide per day, or the combination of furosemide with thiazide diuretics or an aldosterone antagonist [71, 72].

**Table 6:** Risk factors for AKI development in heart failure

Reference	Author, year	Independent risk factors													
		Aging	Diabetes	Cardiac dysfunction	Cerebrovascular disease	CKD (renal dysfunction)	Proteinuria	NGAL elevation	NT-pro-BNP elevation	Diuretics dose/resistance	Hypertension (> 160 mmHg)	Hypotension (< 90 mmHg)	Hyponatremia (< 130 mmol/L)	≥ 3 admissions for AHF	Hemoconcentration by diuretics
[70]	Amin et al. 2010	○	○	○	○	○									
[71]	Wang et al. 2013	○		○		○	○			○		○	○	○	
[72]	Zhou et al. 2012	○	○	○		○				○	○				
[73]	Soyler et al. 2015							○							
[422]	Testani et al. 2010														○
[75]	Aghel et al. 2010							○							
[423]	Pfister et al. 2010		○						○						
[76]	Belziti et al. 2010	○				○						○			

*CKD* chronic kidney disease, *BNP* brain-type natriuretic peptide

○ indicates independent risk factors by multivariable analysis

### Literature review

PubMed was searched for relevant studies published between March 2011 and December 2015, and papers related to the present CQ were identified from the search results. The literature published before March 2011 was referenced from the KDIGO Clinical Practice Guideline for AKI.

## CQ3-4: What should be assessed as risk factors for AKI development in sepsis?

*Recommendation*: Pre-existing renal dysfunction, aging, and the use of renin-angiotensin-aldosterone system inhibitors should be assessed as risk factors.

*Strength of recommendation*: 2

*Quality of evidence*: C

### Summary of evidence

In six observational studies that examined the risk of AKI development in sepsis, pre-existing renal dysfunction, aging, and the use of renin-angiotensin-aldosterone system inhibitors were found to be associated with AKI development in sepsis.

### Commentary

#### Background

Sepsis patients develop AKI frequently [34, 77]. As AKI is associated with a significantly increased mortality [78], it is crucial to assess the risk of its development in sepsis patients. In our search for observational studies aimed at identifying the risk factors for AKI development in sepsis, the numbers of relevant studies and of patients did not make for sufficiently strong evidence; however, several clinical background factors were identified as risk factors for AKI development (Table 7).

**Table 7:** Risk factors for AKI development in sepsis

Reference	Author, year	Pre-existing renal dysfunction	Aging	RAAS inhibitors	Diabetes	Intra-abdominal infection	Blood product	Shock
[80]	Plataki et al. 2011	○	–	○	×	○	○	–
[77]	Suh et al. 2013	○	○	○	×	–	–	○
[79]	Poukkanen et al. 2013	○	–	–	–	–	–	–
[81]	Medeiros et al. 2015	△	○	–	○	–	–	–
[83]	Chang et al. 2012	–	–	–	○	–	–	–
[84]	Venot et al. 2015	–	–	–	×	–	–	–

*RAAS* renin-angiotensin-aldosterone system

○ risk, △ risk without significance, × not risk, − not evaluated

#### Pre-existing renal dysfunction

Pre-existing renal dysfunction is known as a risk factor for AKI development in a variety of pathologies, including sepsis. In an observational study of 992 sepsis patients by Suh et al., 57.7% of the patients developed AKI; one of the risk factors for AKI was renal dysfunction, which was defined as an eGFR < 60 mL/min/1.73 m^2^ (odds ratio 2.398, 95% confidence interval [CI] 1.301–4.420) [77]. In an observational study of 423 patients, Poukkanen et al. also identified pre-existing renal dysfunction as a strong risk factor for AKI in sepsis (odds ratio 7.24, 95% CI 2.36–22.23) [79]. Moreover, Plataki et al. reported that the incidence of AKI was significantly low in individuals with a higher baseline eGFR [80]. In addition, despite the small number of patients and the lack of a significant difference, in an observational study, Medeiros et al. reported that pre-existing renal dysfunction tended to increase the risk of AKI development [81]. Therefore, whenever possible, patients must be examined for pre-existing renal dysfunction when sepsis develops. In addition, when treating septic patients with pre-existing renal dysfunction, it is necessary to monitor the renal function carefully.

#### Aging

Japanese society is aging rapidly. Aging is an underlying cause of age-related organ dysfunction, which creates various medical issues. Suh et al. reported that the risk of AKI development in sepsis increased with age (odds ratio 1.028, 95% CI 1.016–1.041) [77]. Medeiros et al. reported a similar result in an observational study that found AKI to be significantly more frequent in septic patients aged over 65 (odds ratio 1.28, 95% CI 1.12–1.89) [81]. In addition, although the risk was not assessed in a logistic regression analysis, another observational study reported that AKI patients were of a significantly higher age [82]. Therefore, the potential development of AKI must be considered while treating elderly patients with sepsis.

#### Renin-angiotensin-aldosterone system inhibitors

An increase in patients with hypertension has led to a corresponding increase in the number of patients using renin-angiotensin-aldosterone system inhibitors. As these drugs reduce the systemic blood pressure and dilate the efferent arterioles, they may enhance the reduction of the GFR during shock. Therefore, there is a concern that renin-angiotensin-aldosterone system inhibitors may exacerbate the risk of AKI development. In two observational studies that examined whether the use of renin-angiotensin-aldosterone system inhibitors was a risk factor for AKI development in sepsis, the risk was found to be approximately twice as high when using these drugs than when not using them [77, 80]. Therefore, when sepsis develops, the careful monitoring of potential AKI development is recommended in patients using renin-angiotensin-aldosterone system inhibitors. However, there have been no intervention trials to determine whether the withdrawal of these drugs during sepsis can prevent the development of AKI. This question needs to be examined in a RCT.

#### Other risk factors

In addition to the above risk factors, observational studies have also assessed obesity, comorbid diabetes, intra-abdominal bacterial infection, the use of blood products, and hypotension as potential risk factors for AKI development [77, 80, 81, 83]. However, no definitive conclusions have been reached. For instance, one study found that diabetes is not associated with the development of AKI in sepsis [84]. Further evidence needs to be collected in order to determine whether these factors increase the risk of AKI development in sepsis.

### Literature review

PubMed was searched for relevant studies published between March 2011 and December 2015, and papers related to the present CQ were identified from the search results. The literature published before March 2011 was referenced from the KDIGO Clinical Practice Guideline for AKI.

## CQ4-1: Should hospital-acquired AKI and community-acquired AKI be differentiated?

*Recommendation*: Hospital-acquired AKI has a worse survival prognosis than community-acquired AKI. In addition, the relationship between the severity and the mortality may differ between the two types of AKI. Therefore, we suggest that they should be differentiated from one another.

*Strength of recommendation*: Not graded

*Quality of evidence*: D

### Summary of evidence

In a meta-analysis of eight observational studies, the mortality was significantly higher in hospital-acquired AKI than in community-acquired AKI (odds ratio 2.79, 95% confidence interval 2.18–3.56). In studies that used the RIFLE or KDIGO criteria, community-acquired AKI featured a high rate of stage 3 AKI, while hospital-acquired AKI featured a high rate of stage 1 AKI.

### Commentary

Acute kidney injury (AKI) is primarily treated with conservative therapies, such as the optimization of fluid volume or blood pressure and the avoidance of nephrotoxins; in addition, identification of the cause of the kidney injury is recommended. Therefore, it is crucial to recognize the risk factors for AKI and take steps to prevent it in order to improve its outcomes [3].

Acute kidney injury encompasses a broad spectrum of diseases and can occur in the hospital or in the community. However, although community-acquired AKI occurs frequently in low- and middle-income countries which account for roughly 85% of the world population [3], 80–90% of studies have examined hospital-acquired AKI in high-income countries [34]; few studies have compared hospital-acquired and community-acquired AKI. Hospital-acquired AKI is frequently caused by ischemia, nephrotoxins, and sepsis [85], while community-acquired AKI has been found to frequently derived from preventable causes such as dehydration, infection, and childbirth [86]. To determine the state of community-acquired AKI in low- and middle-income countries, the multinational 0by25 initiative conducted the global snapshot study in 2015 [87].

**Fig. 1:**
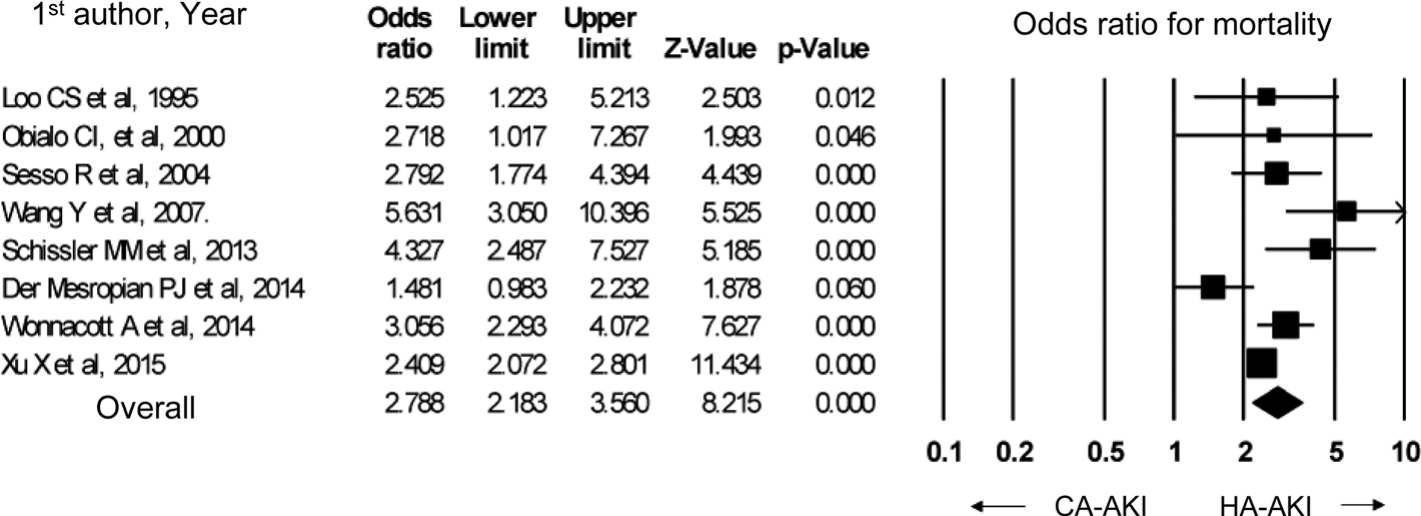
In-hospital mortality in CA-AKI vs HA-AKI. CA-AKI: community-acquired acute kidney injury, HA-AKI: hospital-acquired acute kidney injury

**Fig. 2:**
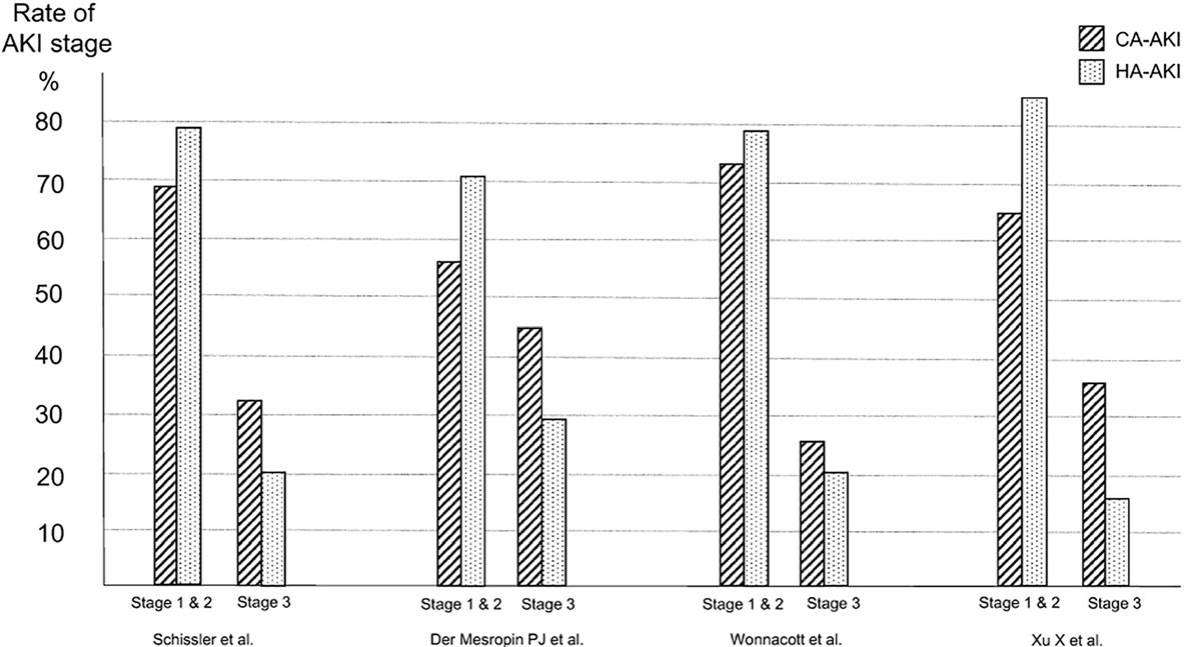
Rate of AKI stage in CA-AKI vs HA-AKI. CA-AKI: community-acquired acute kidney injury, HA-AKI: hospital-acquired acute kidney injury

In order to develop the present guideline, PubMed was used to identify papers that compared hospital-acquired and community-acquired AKI. Eight observational studies were identified [88–95]; among them, two defined AKI based on the RIFLE criteria [92, 93], two used the KDIGO criteria [94, 95], and the other four were published before the RIFLE and KDIGO criteria were proposed [88–91]. Four studies were conducted in high-income countries [89, 92–94], while the other four were conducted in low- and middle-income countries [88, 90, 91, 95]. In all of these studies, community-acquired AKI was associated with a lower mortality (Fig. 1) and shorter hospitalization duration. Moreover, the percentages of patients at each AKI stage (i.e., degree of severity) in the studies that used the AKIN criteria indicated that in all four studies, community-acquired AKI was more severe (i.e., with low percentages of stages 1 and 2 AKI and high percentages of stage 3 AKI), while hospital-acquired AKI showed higher percentages of mild cases (Fig. 2).

Thus, the above-cited studies demonstrate that hospital-acquired AKI and community-acquired AKI have different clinical pictures, as shown in Table 8. The relationship between the severity and mortality may differ between hospital-acquired AKI and community-acquired AKI; therefore, we suggest that they are discriminated from one another.

**Table 8:** Differences between CA-AKI and HA-AKI

	Hospital-acquired AKI	Community-acquired AKI
Mortality	High	Low
Severity	Stage 1 and 2 > stage 3	Stage 3 > stage 1 and 2

However, all the studies used were conducted outside Japan. A further investigation comparing hospital-acquired AKI and community-acquired AKI in Japan is necessary.

### Literature review

PubMed was searched for relevant studies published up to December 2015, and papers that compared hospital-acquired and community-acquired AKI were identified from the search results.

## CQ4-2: Should septic AKI and non-septic AKI be discriminated from each other?

*Recommendation*: Septic AKI may lead to a higher mortality than non-septic AKI; therefore, we suggest that they should be discriminated from each other.

*Strength of recommendation*: Not graded

*Quality of evidence*: D

### Summary of evidence

In a meta-analysis based on nine observational studies, compared to non-septic AKI, septic AKI resulted in a higher in-hospital mortality (odds ratio 2.48, 95% confidence interval 1.76–3.49) and a higher ICU mortality (odds ratio 1.60, 95% confidence interval 1.52–1.69). Although studies that assessed the in-hospital mortality featured publication bias, no such bias was observed related to ICU mortality.

### Commentary

In a report of a large-scale prospective observational study conducted at 54 centers in 23 countries [34], the cause of acute kidney injury (AKI) in the intensive care unit (ICU) was a septic shock in 47.5% of cases and cardiogenic shock in 26.9% of cases. In a large-scale multinational multicenter prospective observational study published in 2015 [96], AKI occurred in 57.3% of ICU patients; the cause of AKI was sepsis in 40.7% of patients and cardiogenic shock in 13.2% of patients. In Japanese epidemiology, the Diagnosis Procedure Combination (DPC) database has been used to examine AKI patients who underwent continuous renal replacement therapy (CRRT) [97]. Among these patients, the most common causes of AKI were cardiovascular disease and other medical diseases, which accounted for approximately half of the patients, followed by sepsis and cardiovascular surgery; compared to all other causes, mortality was low only for cardiovascular surgery.

In developing the present guideline, PubMed was used to identify papers which compared septic and non-septic AKI. Nine observational studies were identified [78, 98–105]; seven of these studies were prospective, while two were retrospective. One of these studies was a retrospective study by Bagshaw et al. which utilized the Australian and New Zealand Intensive Care Society (ANZICS) database [78]; the study featured 14,039 septic AKI patients and 29,356 non-septic AKI patients, a prominently large number of patients compared to other studies. Seven studies compared in-hospital mortality [78, 98–103], while five studies compared ICU mortality [78, 100, 101, 104, 105].

As for AKI diagnostic criteria, six studies used the RIFLE criteria [78, 99–103]; the remaining three studies [98, 104, 105] were published before the RIFLE criteria were proposed. Percentages of patients by RIFLE criteria severity were listed in four studies [78, 99, 100, 102]; Risk was the most common level of severity in one study [100], while Injury was the most common in two studies [78, 99], and Failure was the most common in one study [102]. Causes of sepsis were demonstrated in two studies [99, 101]; in these studies, sepsis was caused by intrathoracic infections (such as pneumonia) and intra-abdominal infections in approximately 30 and 25% of cases, respectively, thus accounting for more than half of all cases. The severities of patients’ illnesses were assessed with the APACHE II, SAPS, and SAPS II severity scores in eight studies [78, 98–101, 103–105]; in all of these studies, septic AKI was more severe than non-septic AKI.

**Fig. 3:**
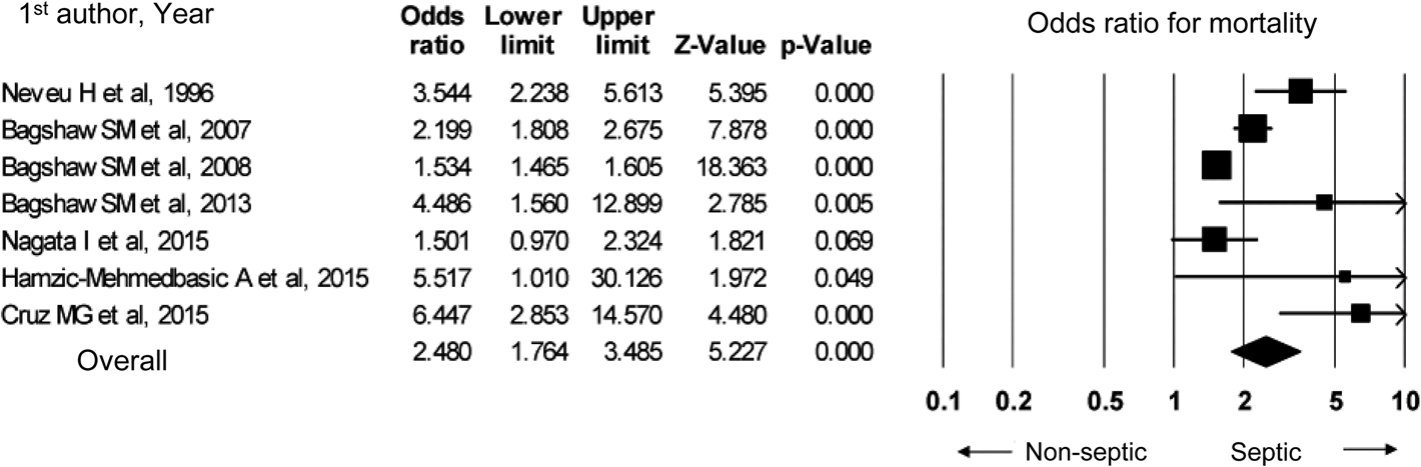
In-hospital mortality in septic AKI vs non-septic AKI

Figure 3 shows the results of a meta-analysis of seven studies which compared in-hospital mortality. In-hospital mortality and ICU mortality may both be higher for septic AKI than for non-septic AKI; therefore, we suggest that the two forms of AKI be discriminated from each other. Septic AKI should be handled in specific ways, such as the admission of patients to the ICU depending on severity, consideration of hemodynamic monitoring, and maintaining fluid volume and renal perfusion pressure.

### Literature searches

Searches were conducted on PubMed for literature published up to November 2015. Papers which compared septic and non-septic AKI were identified from the search results.

## CQ4-3: Should renal AKI and pre-renal AKI be differentiated?

*Recommendation*: The in-hospital mortality may be higher in renal AKI than in pre-renal AKI; therefore, we suggest that they should be differentiated from one another.

*Strength of recommendation*: Not graded

*Quality of evidence*: D

### Summary of evidence

In a meta-analysis of ten observational studies, the in-hospital mortality from renal AKI was higher than that from pre-renal AKI (odds ratio 3.63, 95% confidence interval 1.68–7.83). A significant publication bias was present.

### Commentary

Acute kidney injury (AKI) is classified as either pre-renal, renal (intrinsic), or post-renal. Pre-renal AKI is considered as azotemia resulting from a decreased renal perfusion pressure; conceptually, it is a form of renal impairment with no renal tissue damage, in which the renal function can recover rapidly with early treatment. There are two conceivable approaches to the differentiation of pre-renal AKI from renal AKI. The first approach is to comprehensively assess whether the AKI is pre-renal or renal based on an assessment of the cause of the AKI, hemodynamics, and urinalyses with measuring factors such as the body weight change, vital signs, urine osmolality, fractional excretion of sodium (FENa), fractional excretion of urea nitrogen (FEUN), and urinary sediment. The second approach is to determine whether the renal function recovers immediately after fluid resuscitation. If the renal function recovers within 2–3 days after appropriate fluid resuscitation, the AKI is considered to be volume-responsive, which allows for clinical classification as pre-renal AKI. If the renal function does not recover despite fluid resuscitation, the AKI is considered to be volume-unresponsive, which corresponds to renal AKI. However, when a continued or prolonged reduced renal perfusion pressure results in renal parenchymal injury, or when the reduced renal perfusion pressure is accompanied by a low cardiac output, sepsis, or liver failure, the renal function does not necessarily recover with fluid resuscitation alone [106]. Therefore, even if the AKI is initially assessed as pre-renal, a second test should be performed within 3 days. However, even if the AKI is assessed as pre-renal, a mild elevation in the urinary biomarkers can sometimes be suggestive of a renal tissue injury [107]. As AKI is known to be involved in injuries to multiple organs, including the heart and lungs, even pre-renal AKI may affect the survival prognosis.

Many studies have reported that the in-hospital mortality is lower in volume-responsive AKI—in which the renal function recovers within 3 days of intervention—than in volume-unresponsive AKI. In a recent AKI cohort study of 283 patients in intensive care units (ICUs) at multiple hospitals, the in-hospital mortality rates for non-AKI, volume-responsive AKI, and renal AKI were 23.8, 29.6, and 38.9%, respectively; thus, renal AKI showed the worst outcomes [108]. However, in a study that evaluated AKI based on its underlying causes at diagnosis, the in-hospital mortality was 27.3% in pre-renal AKI versus 19.3% in intrinsic AKI; although the difference was not significant, pre-renal AKI tended to have worse outcomes [109].

**Fig. 4:**
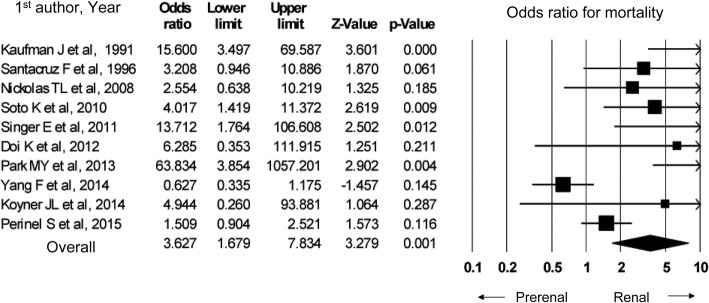
In-hospital mortality in renal AKI vs pre-renal AKI

To develop the present guideline, PubMed was used to identify papers that compared renal and pre-renal AKI in order to assess the difference in the survival outcomes. Ten cohort studies were identified; among them, three differentiated between pre-renal and renal AKI based on the underlying causes of AKI and the urine findings at diagnosis [109–111], while seven differentiated between pre-renal and renal AKI based on the volume responsiveness [107, 108, 112–116]. In our meta-analysis, the in-hospital mortality was significantly higher in renal AKI than in pre-renal AKI (Fig. 4).

Based on the above, pre-renal AKI and renal (intrinsic) AKI have different survival outcomes; therefore, we suggest that they should be distinguished from one another.

### Literature review

PubMed was searched for relevant studies published up to August 2015, and papers related to the present CQ were identified from the search results.

## CQ5-1: Should urinary biomarkers be used for the early diagnosis of AKI?

*Recommendation*: Due to their potential utility in the early diagnosis of AKI, we suggest measuring the urinary NGAL and L-type fatty acid-binding protein (L-FABP). However, the utility of the urinary cystatin C is limited; therefore, we cannot make a recommendation about its use.

*Urinary NGAL and urinary L-FABP*:

*Strength of recommendation*: 2

*Quality of evidence*: B

*Urinary cystatin C*:

*Strength of recommendation*: Not graded

*Quality of evidence*: C

### Summary of evidence

Multiple systematic reviews/meta-analyses have found the urinary NGAL and L-FABP to serve as useful markers for the early diagnosis of AKI. However, future clinical trials that compare AKI interventions based on the conventional diagnostic method using the serum creatinine levels with those based on diagnoses made with urinary biomarkers are necessary to examine whether novel urinary biomarkers are truly useful for the diagnosis of AKI.

Only one systematic review/meta-analysis has assessed the utility of the urinary cystatin C; therefore, firm conclusions as to its utility for the early diagnosis of AKI cannot be made.

### Commentary

The pathological condition previously recognized as acute renal failure (ARF) is now broadly understood to pose a risk of death at an earlier or milder stage than failure. This has prompted a paradigm shift from ARF to acute kidney injury (AKI). However, with the present method of diagnosis, which is based on the identification of an increased level of serum creatinine (sCr) and a reduced urine output, interventions are often mistimed; therefore, there is an urgent need for the clinical application of more sensitive biomarkers. The early diagnosis of AKI enables earlier consultation with a nephrologist, appropriate management of the renal hemodynamics, and the avoidance of exposure to nephrotoxins. Therefore, we examined whether urinary biomarkers should be used for the early diagnosis of AKI based on a relatively large number of studies on AKI in adult patients having received cardiovascular surgery and those in intensive care units (ICUs).

Neutrophil gelatinase-associated lipocalin (NGAL) is a low molecular weight protein (molecular weight, approx. 25,000) that belongs to the lipocalin protein family and is secreted by activated neutrophils. In addition to inducing kidney development and possessing renoprotective and antibacterial effects, NGAL is also expressed in the distal nephron in kidney injury. Multiple systematic reviews/meta-analyses have found the urinary NGAL to be useful for the early diagnosis of AKI [117–122]. Among the studies cited in these systematic reviews/meta-analyses, 16 (for a total of 2194 patients) were related to the present CQ (Table 9) [123–138]. The subjects consisted of patients who had undergone cardiovascular surgery (14 studies, 1531 patients in all) [123, 124, 126–130, 132–138] and ICU patients (two studies, 663 patients in all) [125, 131]. The majority of these studies defined AKI according to the RIFLE or AKIN criteria (i.e., an increase in sCr) or to criteria conforming to the RIFLE or AKIN ones. A total of 549 patients (25%) were diagnosed with AKI. In an assessment of the early diagnostic capacity of the urinary NGAL over the 6-h period immediately after surgery or ICU admission, the area under the receiver operating characteristic curve (AUC) was 0.50–0.98 (0.77 with an unweighted mean). In 75% (12/16) of the studies, the AUC was ≥ 0.70, thus showing moderate or better diagnostic accuracy; therefore, the urinary NGAL was found to be useful for the early diagnosis of AKI. However, the clinical studies related to the present CQ raised several issues about the clinical application of the urinary NGAL, including the following: some studies did not use officially approved measurement methods; multiple measurement methods were used, and they were not standardized; there was no set cutoff value; urinary tract infections and urologic diseases increase the urinary NGAL levels [139]; and there are very few relevant clinical studies of Japanese subjects.

**Table 9:** Urinary NGAL for early AKI diagnosis

Reference	Author, year	Cohort	All case (*n*)	AKI case		AKI definition		Urine sampling	Cutoff	Sensitivity	Specificity	AUC (95% CI)
				*n*	%	sCr criteria	UO criteria					
[123]	Koyner et al. 2008	Cardiac surgery	72	34	47	Increase ≥ 25% in 3 days OR RRT	N/A	ICU admission	572 ng mgCr^−1^	0.61	0.73	0.71 (0.58–0.83)
[124]	Tuladhar et al. 2009	Cardiac surgery	50	9	18	Increase ≥ 0.5 mg/dL in 48 h	N/A	2 h postoperative	393 ng mmolCr^−1^	0.93	0.78	0.96 (0.90–1.00)
[138]	Paarmann et al. 2013	Cardiac surgery	136	29	21	AKIN ≥ 1	N/A	6 h postoperative	NR	NR	NR	0.61 (0.48–0.73)
[125]	de Geus et al. 2011	ICU	632	171	27	RIFLE≥ R	N/A	ICU admission	NR	NR	NR	0.80
[126]	Wagener et al. 2006	Cardiac surgery	81	16	20	RIFLE ≥ R	N/A	18 h postoperative	213 ng ml^−1^	0.73	0.78	0.80 (0.57–1.03)
[127]	Wagener et al. 2008	Cardiac surgery	426	85	20	AKIN ≥ 1	N/A	18 h postoperative	65 ng ml^−1^	0.39	0.78	0.61 (0.54–0.68)
[128]	Xin et al. 2008	Cardiac surgery	33	9	27	AKIN ≥ 1	AKIN ≥ 1	2 h postoperative	250 μg mmolCr^−1^	0.81	0.78	0.93
[129]	Han et al. 2009	Cardiac surgery	90	36	40	Increase ≥ 0.5 mg/dL in 72 h	N/A	3 h postoperative	456 ng mgCr^−1^	0.71	0.39	0.65 (0.54–0.76)
[130]	Liangos et al. 2009	Cardiac surgery	103	13	13	Increase ≥ 50% in 72 h	N/A	2 h post-CPB	166 ng mgCr^−1^	0.67	0.11	0.50 (0.33–0.68)
[131]	Makris et al. 2009	ICU	31	11	35	RIFLE ≥ R	N/A	ICU admission	25 ng ml^−1^	0.91	0.95	0.98 (0.82–0.98)
[132]	Heise et al. 2011	Cardiac surgery	50	38	76	AKIN ≥ 1	AKIN ≥ 1	6 h after ICU admission	16.8 μg L^−1^	0.82	0.78	0.77 (0.63–0.88)
[133]	Ejaz et al. 2012	Cardiac surgery	100	27	27	AKIN ≥ 1	N/A	24 h after operation	NR	NR	NR	0.62 (0.49–0.75)
[134]	Sargenti et al. 2012	Cardiac surgery	52	15	29	AKIN ≥ 1	AKIN ≥ 1	4 h postoperative	55.2 μg gCr^−1^	0.55	0.73	0.71 (0.56–0.85)
[135]	Liebetrau et al. 2013	Cardiac surgery	141	19	13	KDIGO ≥ 2	KDIGO ≥ 2	4 h postoperative	NR	NR	NR	0.90 (0.81–0.99)
[136]	Liu et al. 2013	Cardiac surgery	109	26	24	AKIN ≥ 1	N/A	2 h postoperative	33.73 ng mgCr^−1^	0.81	0.83	0.87 (0.78–0.97)
[137]	Munir et al. 2013	Cardiac surgery	88	11	13	AKIN ≥ 1	AKIN ≥ 1	4 h postoperative	87 ng ml^−1^	0.91	0.99	0.91 (0.83–0.96)

*sCr* serum creatinine, *UO* urine output, *RRT* renal replacement therapy, *AUC* area under the curve, *95% CI* 95% confidence interval, *NR* not reported

The L-type fatty acid-binding protein (L-FABP) is a low molecular weight protein (molecular weight, approx. 14,000) localized in the cytoplasm of human renal proximal tubular cells. By binding to free fatty acids and transporting them to mitochondria and peroxisomes, the L-FABP promotes beta-oxidation, contributes to energy production, and helps to maintain homeostasis. When the proximal tubule is subjected to ischemia or oxidative stress, the expression of the L-FABP is enhanced and its urinary excretion increases. The urinary L-FABP has been demonstrated to be useful for the early diagnosis of AKI [117, 118, 140] by multiple systematic reviews/meta-analyses. Among the studies cited in these systematic reviews/meta-analyses, seven (for a total of 2416 patients) were related to the present CQ (Table 10) [136, 141–146]. The subjects consisted of patients who had undergone cardiovascular surgery (three studies, 271 patients in all) [136, 141, 146] and ICU patients (four studies, 2145 patients in all) [142–145]. These studies generally defined AKI according to the RIFLE or AKIN criteria (i.e., an increase in sCr). A total of 298 patients (12%) were diagnosed with AKI. In an assessment of the early diagnostic capacity of the urinary L-FABP over the 12-h period immediately after surgery or ICU admission, the AUC was 0.70–0.95 (0.81 with an unweighted mean). In all seven studies, the AUC was ≥ 0.70, thus showing moderate or better diagnostic accuracy; therefore, the urinary L-FABP was found to be useful for the early diagnosis of AKI. However, the timing of the urinary L-FABP measurement must be chosen carefully according to the different AKI etiologies. Measuring reagents for the L-FABP are available in Japan; these are standardized and covered by public health insurance.

**Table 10:** Urinary L-FABP for early AKI diagnosis

Reference	Author, year	Cohort	All case (*n*)	AKI case		AKI definition		Urine sampling	Cutoff	Sensitivity	Specificity	AUC (95% CI)
				*n*	%	sCr criteria	UO criteria					
[146]	Katagiri et al. 2012	Cardiac surgery	77	28	36	AKIN ≥ 1	No	12 h postoperative	51.6 ng ml^−1^	0.64	0.79	0.76 (0.62–0.86)
[141]	Matsui et al. 2012	Cardiac surgery	85	48	56	AKIN ≥ 1	No	0 h postoperative	54.6 ng mgCr^−1^	0.77	0.92	0.86 (0.78–0.94)
[142]	Doi et al. 2011	ICU	339	66	19	RIFLE ≥ R	No	Within 12 h ICU admission	NR	NR	NR	0.80 (0.73–0.86)
[143]	Matsui et al. 2011	ICU	26	14	54	AKIN ≥ 1	No	ICU admission	44.1 μg gCr^−1^	0.86	1.00	0.95
[144]	Cho et al. 2013	ICU	145	54	37	AKIN ≥ 1	No	ICU admission	28.45 ng ml^−1^	0.72	0.76	0.78 (0.70–0.86)
[136]	Liu et al. 2013	Cardiac surgery	109	26	24	AKIN ≥ 1	No	0 h postoperative	2226.5 μg gCr^−1^	0.85	0.82	0.84
[145]	Nickolas et al. 2012	ICU	1635	62	4	RIFLE ≥ R	No	ED visit	12.9 ng ml^−1^	0.50	0.80	0.70 (0.65–0.76)

*sCr* serum creatinine, *UO* urine output, *RRT* renal replacement therapy, *AUC* area under the curve, *95% CI* 95% confidence interval, *NR* not reported

Cystatin C is a low molecular weight protein (molecular weight, approx. 13,000) produced by nucleated cells all over the body that inhibits the cell injury caused by cysteine proteases. After being secreted outside the cells, cystatin C is filtered by the glomerulus; 99% of it is then absorbed by the proximal tubule and catabolized. Therefore, tubular injuries are affected by the reabsorption of cystatin C; consequently, the cystatin C concentration in urine has been examined as a biomarker of AKI. There has been one systematic review/meta-analysis of the early diagnostic capacity of the urinary cystatin C [147]; the latter cited six studies [116, 123, 130, 132, 148, 149]. In a pool analysis of four studies that used urinary creatinine-adjusted data [116, 130, 148, 149], the sensitivity for the early diagnostic capacity of urinary cystatin C was 0.52, the specificity was 0.70, and the AUC was 0.64 (95% confidence interval 0.62–0.66); therefore, the diagnostic accuracy was low, indicating that the urinary cystatin C is of limited utility for the early diagnosis of AKI. The measurement of cystatin C in urine samples is not covered by public health insurance in Japan. Although the urinary albumin has been reported to be a potential biomarker of early AKI [150], the number of relevant studies is limited; thus, the utility of the urinary albumin as a biomarker remains unknown.

As described above, the urinary NGAL and L-FABP have been identified as useful biomarkers for the early diagnosis of AKI. However, future clinical trials that compare AKI interventions based on the conventional diagnostic method using the serum creatinine levels with those based on diagnoses made with urinary biomarkers are necessary to examine whether novel urinary biomarkers are truly useful for the diagnosis of AKI.

### Literature review

PubMed was searched for relevant studies published up to August 2015, and papers related to the present CQ were identified from the search results.

Search query: (((“acute kidney injury”[MeSH Terms] OR “acute kidney injury”[tw] OR “acute renal failure”[tw]) AND (“biological markers”[MeSH Terms] OR “biological markers”[All Fields] OR “biomarker”[All Fields])) AND (“diagnosis”[Subheading] OR “diagnosis”[All Fields] OR “diagnosis”[MeSH Terms])) AND (Meta-Analysis[PT] OR systematic[SB]).

## CQ5-2: Should urinary biomarkers be used to predict the AKI severity and mortality?

*Recommendation*: Although the urinary NGAL is of limited utility in predicting the AKI severity and mortality, we suggest measuring urinary NGAL. The utilities of the urinary L-FABP and cystatin C in this regard are unclear.

*Urinary NGAL*:

*Strength of recommendation*: 2

*Quality of evidence*: C

*Urinary L-FABP and urinary cystatin C*:

*Strength of recommendation*: Not graded

*Quality of evidence*: D

### Summary of evidence

Multiple systematic reviews/meta-analyses about the use of the urinary NGAL to predict the AKI severity and survival outcomes have suggested that the urinary NGAL is potentially useful, albeit limitedly, in predicting the severity in relation to death and renal replacement therapy initiation. The number of studies on the urinary L-FABP and cystatin C is limited; therefore, their utilities in predicting the AKI severity in relation to death and renal replacement therapy initiation are unclear.

### Commentary

Acute kidney injury (AKI) has been shown to be involved not only in short-term kidney injury but also in the subsequent renal outcomes and mortality. Therefore, the prediction of these outcomes is of clinical importance. In addition to the serum creatinine (sCr), the cystatin C, and the estimated glomerular filtration rate (eGFR), all of which reflect the renal function, studies have examined the urinary NGAL, NAG, L-FABP, and cystatin C as biomarkers of kidney injury [117–119, 121, 147, 151]. The results of several systematic reviews and meta-analyses of these studies have been reported in recent years.

Regarding the utility of the urinary NGAL for the prediction of the renal outcomes and mortality, multiple systematic reviews/meta-analyses have indicated that the urinary NGAL could help to predict the AKI severity in relation to death and the initiation of renal replacement therapy (RRT). In a meta-analysis of nine studies (a total of 1948 patients), the odds ratios for RRT requirement and in-hospital mortality based on increased urinary and serum NGAL levels were 12.9 and 8.8, respectively [122]. Meanwhile, in an analysis of 13 studies (a total of 1079 patients) on the recovery of the renal function after kidney transplantation, the NGAL was shown to be a useful predictor of AKI, with an area under the curve (AUC) of receiver operating characteristic (ROC) of 0.87 [120]. However, these meta-analyses examined both urine and blood specimens; therefore, the results should be interpreted cautiously. Another cautionary point is that reports of NGAL measurements have used multiple measurement kits with different upper and lower limits.

In a meta-analysis of studies that used the urinary L-FABP to predict the need for RRT (three studies, 436 patients in all) and the in-hospital mortality (three studies, 561 patients in all), while no significant difference was observed in the need for RRT, the in-hospital mortality odds ratio was 13.7 (*p* = 0.008) [140]. Although measurement of the urinary L-FABP is covered by public health insurance in Japan, in principle, it can only be calculated once every 3 months. In addition, the urinary L-FABP has been reported to be elevated in patients with diabetes and chronic kidney diseases.

Regarding the examinations of the cystatin C, a meta-analysis of seven studies (a total of 2941 patients) showed that high levels of cystatin C are a risk factor for death (odds ratio 2.3) [152]. However, both blood and urine samples were examined in this analysis. Although measurement of the serum cystatin C is covered by public health insurance in Japan, that of the urinary cystatin C is not.

Other AKI markers that have been used in the past include the NAG, which increases by the release from the tubular epithelium brush border into the urine, and the β2microglobulin (β2MG) and α1microglobulin (α1MG), which will be increased by impaired tubular epithelial cell reabsorption. However, these markers are fraught with problems, as the samples are unstable (e.g., they are subject to changes in the urinary pH) and they are easily affected by the serum concentration of β2MG and α1MG. In addition, the levels of these markers can be increased by tubular disorders caused by proteinuria associated with glomerular injuries.

In measuring and assessing these urinary biomarkers, one should be cautious about the timing of the sample collection. In surgeries that use cardiopulmonary bypasses, the levels of the urinary NGAL and L-FABP increase 2–6 h after the surgery before declining gradually. Other biomarkers’ levels also peak within a relatively short period and then decline. Therefore, if the timing of the AKI development is unknown, it is necessary to consider whether the testing was performed at the appropriate time for measurement.

### Literature review

PubMed was searched for relevant studies published up to August 2015, and papers related to the present CQ were identified from the search results.

Search query: (((“acute kidney injury”[MeSH Terms] OR “acute kidney injury”[tw] OR “acute renal failure”[tw]) AND (“biological markers”[MeSH Terms] OR “biological markers”[All Fields] OR “biomarker”[All Fields])) AND (“diagnosis”[Subheading] OR “diagnosis”[All Fields] OR “diagnosis”[MeSH Terms])) AND (Meta-Analysis[PT] OR systematic[SB]).

## CQ5-3: Should urinary biomarkers be used to differentiate pre-renal AKI from renal AKI?

*Recommendation*: Although the urinary NGAL is of limited utility for the differentiation of pre-renal AKI from renal AKI, we suggest measuring urinary NGAL. The utilities of the urinary NAG, L-FABP, and cystatin C in this regard are unknown.

*Urinary NGAL*:

*Strength of recommendation*: 2

*Quality of evidence*: C

*Urinary NAG, L-FABP, and cystatin C*:

*Strength of recommendation*: Not graded

*Quality of evidence*: D

### Summary of evidence

Observational studies have reported that the urinary NGAL is mildly elevated in pre-renal AKI and highly elevated in renal AKI; therefore, the urinary NGAL can be useful in the differentiation of pre-renal from renal AKI. However, the measurement points and cutoff values have not yet been determined. Therefore, we recommend incorporating other laboratory findings and physical findings to differentiate pre-renal from renal AKI. The utility of other urinary biomarkers in this regard is unknown.

### Commentary

The conventional indicators for the differentiation of pre-renal acute kidney injury (AKI) and renal AKI include the urine osmolality, the fractional excretion of sodium (FENa), the fractional excretion of urea nitrogen (FEUN), and the urine sediment findings; however, none of these tests can be considered sufficiently sensitive or specific. There have been no systematic reviews or meta-analyses of studies on the use of urinary biomarkers for the differentiation of pre-renal from renal AKI; only a small number of observational studies are available. In multiple studies in which patients diagnosed with AKI were divided into patients with pre-renal or renal AKI, the degree of elevation of the urinary NGAL was found to be potentially useful for the differentiation of these two types of AKI.

In Nickolas et al.’s examination of the urinary NGAL in 635 patients hospitalized after emergency room visits, the mean urinary NGAL level was significantly higher in renal AKI patients (*n* = 30; 416 ± 387 μg/gCr) than in pre-renal AKI patients (*n* = 88; 30.1 ± 92.0 μg/gCr) [112]. In a report of 145 hospitalized patients by Singer et al., the median urinary NGAL level was significantly higher in renal AKI patients (*n* = 75; 255.6 μg/L [98.5–872.9 μg/L]) than in pre-renal AKI patients (*n* = 32; 31.3 μg/L [15.9–75.5 μg/L]) [115]. Moreover, a urinary NGAL cutoff level of 104 μg/L yielded a high sensitivity (0.75), high specificity (0.88), and high positive likelihood ratio (5.97) for the diagnosis of renal AKI. In a report by Seibert et al., the urinary NGAL levels within 3 days of hospital admission were significantly higher in renal AKI patients (458.1 ± 695.3 ng/mL) than in pre-renal AKI patients (64.8 ± 62.1 ng/mL). In addition, a urinary NGAL cutoff level of 52 ng/mL yielded high sensitivity (0.75), high specificity (0.72), and a high area under the receiver operating characteristic curve (AUC 0.89) for the diagnosis of renal AKI [153].

Although one study found that the urinary NGAL levels of pre-renal AKI patients were not elevated [154], other studies have reported that the urinary biomarker levels were mildly but significantly higher in pre-renal AKI patients than in patients without AKI. In a study by Doi et al. in which 129 out of 337 patients who were admitted to the intensive care unit (ICU) were diagnosed with AKI and in which transient AKI (pre-renal AKI) was defined as the recovery of the serum creatinine (sCr) to within 0.3 mg/dL above baseline within 48 h, 51 patients were diagnosed with transient AKI [107]. Upon ICU admission, transient AKI patients’ levels of urinary NGAL, urinary L-FABP, NAG, and urinary albumin were mildly but significantly higher than those of non-AKI patients. Nejat et al. compared the urinary biomarkers upon ICU admission of 285 non-AKI patients, 61 pre-renal AKI patients (with pre-renal AKI defined as a FENa < 1.0% and recovery of the sCr levels within 48 h), and 114 renal AKI patients [155]. The median urinary NGAL levels of non-AKI patients and of pre-renal AKI patients were 7.7 μg/mmolCr (3.3–35 μg/mmolCr) and 14 μg/mmolCr (6.5–56 μg/mmolCr), respectively; thus, pre-renal AKI patients showed a tendency to have a mildly higher urinary NGAL level (*p* = 0.052). In addition, the median urinary NGAL level of renal AKI patients was 44 μg/mmolCr (16–345 μg/mmolCr), which was significantly higher than that of pre-renal AKI patients. The median urinary cystatin C levels of non-AKI patients and pre-renal AKI patients were 0.026 mg/mmolCr (0.010–0.12 mg/mmolCr) and 0.054 mg/mmolCr (0.017–0.53 mg/mmolCr), respectively; thus, the urinary cystatin C was significantly higher in pre-renal AKI patients than in non-AKI patients. The median urinary cystatin C level of renal AKI patients was 0.21 mg/mmolCr (0.05–1.9 mg/mmolCr), which was significantly higher than that of pre-renal AKI patients.

As described above, the urinary NGAL is mildly elevated in pre-renal AKI patients and highly elevated in renal AKI patients, which suggests that the urinary NGAL is potentially useful for the differentiation of pre-renal from renal AKI. However, the measurement points, cutoff values, and the need for urine creatinine correction have not yet been determined; these issues must be considered in the future. Therefore, pre-renal and renal AKI cannot be differentiated based on the urinary NGAL alone; hence, we recommend a comprehensive assessment that also incorporates other laboratory and physical findings.

### Literature review

PubMed was searched for relevant studies published up to November 2015, and papers related to the present CQ were abstracted from the search results.

Search query: “acute kidney injury”[MeSH Terms] OR acute kidney failure[tw] OR acute renal failure[tw] OR acute kidney injury[tw] OR acute kidney injuries[tw] OR acute kidney injury[tw] OR acute kidney injury[tw] OR acute renal injuries[tw] OR acute renal injury[tw] OR acute kidney insufficiencies[tw] OR acute kidney insufficiency[tw] OR acute renal insufficiencies[tw] OR acute renal insufficiency[tw] OR acute tubular necrosis[tw] OR ARI[tw] OR AKI[tw] OR ARF[tw] OR AKF[tw] OR ATN[tw] AND NGAL[tw] OR neutrophil gelatinase-associated lipocalin[tw] OR L-FABP[tw] OR liver-type fatty acid-binding protein[tw] OR NAG[tw] OR N-acetyl-β-D-glucosaminidase[tw]) AND (“pre-renal”[tw] OR “pre-renal azotaemia”[tw] OR prerenal[tw]) NOT (child[tw] OR children[tw] OR infant[tw] OR pediatrics[tw]).

## CQ6-1: Is low-dose atrial natriuretic peptide recommended for prevention or treatment of AKI?

*Recommendation*: Although low-dose atrial natriuretic peptide has been suggested to be useful for prevention of AKI, relevant reports remain insufficient. Evidence of low-dose atrial natriuretic peptide for the treatment of AKI is limited.

*Strength of recommendation*: Not graded

*Quality of evidence*: D

### Summary of evidence

Since atrial natriuretic peptide (ANP) preparation “carperitide” is covered by health insurance for the treatment of congestive heart failure in Japan, we only investigated randomized controlled trials (RCTs) of ANP in which carperitide was used. A 2009 Cochrane review [156] suggested that low-dose ANP may reduce the frequency of renal replacement therapy (RRT) in the setting of AKI prevention. However, the 2012 KDIGO Clinical Practice Guideline for AKI [3] and a 2013 Cochrane review [157] carefully assessed individual pieces of evidence and lead to a revised conclusion that there is insufficient evidence to declare that low-dose ANP is effective for the treatment or prevention of AKI. Regarding 2009 and 2011 reports on cardiovascular surgery which focused on AKI prevention [158, 159], doubts were raised concerning issues such as randomization and blinding methods. In a 2011 paper, administration of low-dose ANP resulted in a significantly reduced rate of RRT after 1 year [159]. Since 2011, there have been two new RCTs related to AKI prevention; however, due to few numbers of patients, we judged these RCTs lack sufficient statistical power. Currently, there is no strong evidence indicating that low-dose ANP is ineffective for the prevention or treatment of AKI, but rather the evidence that does indicate its effectiveness is of insufficient quality.

### Commentary

Atriuretic peptide (ANP) is a circulating hormone that was discovered in Japan. Along with brain natriuretic peptide (BNP) and C-type natriuretic peptide (CNP), they make up the natriuretic peptide family [160–162]. In healthy conditions, ANP is produced from the atria; however, in heart failure, the production and secretion of ANP from both the atria and ventricles are enhanced [162, 163]. ANP possesses multiple independent modes of actions, including vasodilation, inhibition of sodium reabsorption, inhibition of water reabsorption, elevation of glomerular filtration rate via afferent arteriole dilation and efferent arteriole constriction, reduction of renin activity, angiotensin II concentration, aldosterone concentration in the blood, and sympathetic nerve inhibition [164]. Combined together, continuous infusion of ANP or BNP on laboratory animals and humans exerts a powerful natriuretic effect [165]. Therefore, in the prevention or treatment of AKI, ANP is expected to elicit renoprotective effect through diuresis and increase of glomerular filtration rate. Many clinical studies have been carried out in this context. However, administration of high-dose ANP reduces systemic blood pressure, thereby potentially canceling out the abovementioned renoprotective effect. Therefore, it is crucial to identify the optimal dose of ANP for the achievement of renoprotective effect. Based on the 2012 KDIGO Clinical Practice Guideline for AKI, the present guideline defined low-dose ANP as ≤ 50 ng/kg/min and high-dose ANP as ≥ 100 ng/kg/min.

As to the assessment of the therapeutic effect of ANP after the development of AKI, there are two large-scale randomized controlled trials (RCTs) in which more than 200 participants were assigned into two arms. In both RCTs, high-dose ANP (200 ng/kg/min, 24 h) failed to reduce the incidence of RRT [166, 167]. In a later small-scale RCT using low-dose ANP (50 ng/kg/min, mean 127 h), the ANP group exhibited a significant reduction in the frequency of RRT as compared with the placebo group [168]. There have been no subsequent RCTs investigating the therapeutic effects of low-dose ANP for AKI. Therefore, the present guideline could not offer a definitive recommendation.

On the other hand, concerning the prevention of AKI by ANP, 13 RCTs were found (excluding AKI from contrast-induced nephropathy); all of these were Japanese clinical trials which used low-dose ANP. In most of them, the serum creatinine (sCr) values became significantly lower in the ANP group than in the control group. However, based on a strict application of the AKI diagnostic criteria from the 2012 KDIGO Clinical Practice Guideline for AKI [3], we found no studies in which the ANP group demonstrated a significant reduction in the incidence of AKI.

In a 2009 Cochrane review by Nigwekar et al. [156], low-dose ANP was reported to potentially reduce the need for RRT during prevention of AKI in major surgery, particularly cardiovascular surgery. However, the administration of high-dose ANP for AKI treatment was shown to increase the frequency of adverse events such as hypotension and arrhythmia. The 2012 KDIGO Clinical Practice Guideline for AKI and a 2013 Cochrane review by Zacharias et al. [157] judged that the numbers of patients, the details of the randomization and blinding, and the rigor of the endpoint definitions were insufficient in previous studies. Consequently, the effectiveness of low-dose ANP for the prevention of AKI was deemed inconclusive.

Although RCTs conducted in Japan have suggested that ANP is useful for the prevention of AKI, the quality of the research methods used was debatable. Therefore, we conclude that evidence for the effectiveness of low-dose ANP both for the prevention and treatment of AKI is insufficient, making a definitive recommendation impossible. The ANP preparations used in RCTs in Japan and in the West are carperitide (product name: Hamp®) and anaritide, respectively. Although carperitide has been available in Japan for the treatment of congestive heart failure since 1995, its use for the prevention or treatment of AKI is not covered by health insurance. Urodilatin (product name: Ularitide®) is an ANP-related hormone with four amino acid residues added to the N-terminus of ANP; it is produced in the distal nephron [169]. Clinical trials for ularitide have been conducted outside of Japan [170], and we did not mention it in the present guideline.

### Literature review

Searches were conducted for relevant studies published between January 2008 and August 2015. The literature published prior to 2008 was referenced from a 2009 Cochrane review by Nigwekar et al. [156]. All RCTs related to contrast-induced nephropathy were excluded.

## CQ6-2: Are loop diuretics recommended for the prevention and treatment of AKI?

*Recommendation*: We do not recommend loop diuretics for the prevention of AKI. We also suggest that loop diuretics should not be administered for the treatment of AKI, except to correct fluid overload.

*Prevention*:

*Strength of recommendation*: 1

*Quality of evidence*: B

*Treatment*:

*Strength of recommendation*: 2

*Quality of evidence*: C

### Summary of evidence

Previous guidelines and systematic reviews do not recommend the use of loop diuretics for the prevention or treatment of AKI. There have been no new RCTs to contradict the results of previous clinical trials on loop diuretics for AKI.

### Commentary

Loop diuretics inhibit the sodium reabsorption and exert a diuretic effect by inhibiting the Na-K-2Cl cotransporter in the thick ascending limb of the loop of Henle. Due to their theoretical effectiveness against acute kidney injury (AKI), clinical trials involving loop diuretics have long been performed. For example, by ensuring a diuretic effect, loop diuretics can prevent the tubular obstruction induced by cell shedding; in addition, they increase the medullary oxygenation and the renal medullary blood flow.

Three randomized controlled trials (RCTs) have compared the use of loop diuretics to that of a placebo or to standard therapy for the prevention of AKI [171–173]. In a meta-analysis by Ho and Power, loop diuretics failed to yield a significant improvement in the in-hospital mortality or in the percentage of patients who required renal replacement therapy (RRT) [174]. Moreover, although different RCTs have defined AKI differently, none has yet demonstrated a statistically significant reduction in the incidence of AKI in a loop diuretics group. In fact, in an RCT by Lassnigg et al., the loop diuretics group showed an increased incidence of renal dysfunction (14.6 vs 0%, *p* < 0.01) [172]. Based on the above, the present guideline does not recommend the use of loop diuretics for AKI prevention.

Seven RCTs have also compared the use of loop diuretics to that of a placebo or to standard therapy for the treatment of existing AKI [175–181]. In the abovementioned meta-analysis, the loop diuretics group did not demonstrate a significant improvement in the in-hospital mortality or the percentage of patients who required RRT [174]. Although different RCTs have used different definitions of recovery from renal dysfunction, no RCT to date has demonstrated a significant increase in the percentage of patients who recovered from renal dysfunction in the loop diuretics group. Among the abovementioned seven RCTs, two limited their subjects to AKI patients who underwent RRT; in both of these, the loop diuretics group did not demonstrate a significant reduction in the duration of the RRT or in the early recovery from renal dysfunction [180, 181]. In addition, one meta-analysis showed that high-dose furosemide, which is often used to treat AKI, significantly increased symptoms such as tinnitus and temporary deafness (as compared with their incidence in control groups) [182]. Based on the above, the present guideline does not recommend the use of loop diuretics for AKI treatment.

On the other hand, in AKI with a reduced urine output, loop diuretics may help to correct the fluid overload and to improve any electrolyte imbalance (such as hyperkalemia). However, there are currently no RCTs in which loop diuretics were administered specifically to treat AKI with these sorts of clinical manifestations—hence the above suggestion. The guidelines published by the KDIGO and NICE (National Institute for Health and Clinical Excellence) do not exclude the use of loop diuretics for the correction of fluid overload.

As for diuretics other than loop diuretics, RCTs have also examined mannitol for the prevention of AKI. In a meta-analysis by Yang et al., mannitol did not demonstrate evident effectiveness for the prevention of AKI [183]. In subsequent RCTs, the mannitol groups also failed to demonstrate significant improvement in their RRT initiation rates or in-hospital mortality [184, 185].

### Literature review

PubMed was searched for relevant studies published between January 2012 and April 2015, and papers related to the present CQ were identified from the search results. The literature published before January 2012 was referenced from the KDIGO Clinical Practice Guideline for AKI.

## CQ6-3: Is low-dose dopamine recommended to prevent and treat AKI?

*Recommendation*: We recommend not using low-dose dopamine to prevent or treat AKI.

*Strength of recommendation*: 1

*Quality of evidence*: A

### Summary of evidence

The KDIGO Clinical Practice Guideline for AKI suggests not to use low-dose dopamine to prevent or treat AKI. Since the publication of the KDIGO guideline, the efficacy of low-dose dopamine for the prevention of AKI has been examined in five RCTs. None of them have found low-dose dopamine to be effective.

### Commentary

Dopamine has been widely used to treat severely ill patients especially before 2000. The administration of dopamine, particularly low-dose (1–3 μg/kg/min), to healthy individuals is considered to bring increases in renal vasodilation, in natriuresis, and in the glomerular filtration rate (GFR); therefore, low-dose dopamine had been anticipated to have a renoprotective effect. However, many of the clinical studies on dopamine have been found to be of poor quality due to a variety of issues, including small numbers of patients, unsuitable randomization, insufficient statistical powers, and unsuitable outcomes related to the clinical utility. Furthermore, due to the negative results in several randomized controlled trials (RCTs) that applied appropriate statistical powers and sample sizes, the use of dopamine is less commonly recommended today [186]. In addition, the renal vasodilation effect observed in healthy individuals has been found not to occur in acute kidney injury (AKI) patients [187].

However, there is only limited evidence of harm caused by the use of low-dose dopamine to prevent or treat AKI. Although a 2005 meta-analysis by Friedrich et al. [188] did not find low-dose dopamine to significantly increase the incidence of adverse effects, there are many literatures related to the adverse effects of dopamine. The potential adverse effects of dopamine include tachycardia, myocardial ischemia, a reduced intestinal blood flow, hypopituitarism, and the inhibition of the T cell function.

Friedrich et al. conducted a meta-analysis of studies in which low-dose dopamine was used to treat or prevent AKI [188]. Their analysis of 61 randomized and semi-randomized trials determined that low-dose dopamine did not prolong survival, reduce the rate of dialysis initiation, or improve the renal function; in addition, the urine output was found to be only improved on the day the dopamine treatment was initiated. Based on the absence of positive studies about the use of dopamine to prevent and treat AKI, and in consideration of the information about the previously described adverse effects of dopamine, the 2012 KDIGO Clinical Practice Guideline for AKI recommends that low-dose dopamine should not be used to prevent or treat AKI (1A).

For the present CQ, we searched the literature to retrieve new evidence that has emerged since the publication of the KDIGO guideline. The literature review and assessment of the abstracts revealed five trials that potentially contained new evidence not included in the existing meta-analyses [189–193]. The subjects were heart failure patients in three trials, laparoscopic surgery patients in one trial, and severe obstructive jaundice patients in one trial. In the three trials involving heart failure patients, dopamine failed to improve the outcomes. The trials involving laparoscopic surgery patients and severe obstructive jaundice patients did not examine any clinically useful outcomes.

Based on the above-described KDIGO guideline recommendation and on the fact that no subsequent trials have demonstrated low-dose dopamine to be effective in the prevention or treatment of AKI, we offer the same quality of evidence and strength of recommendation as the KDIGO guideline.

### Literature review

PubMed was searched for relevant studies published between December 24, 2009, and December 22, 2014, using “dopamine”, “AKI”, and “RCT” as search terms.

Search query: ((“dopamine”[MeSH Terms] OR “dopamine”[All Fields]) AND ((“kidney”[MeSH Terms] OR “kidney”[All Fields]) OR renal[All Fields]) AND low[All Fields]) AND (Randomized Controlled Trial[ptyp] AND “2009/12/24”[PDAT]:“2014/12/22”[PDAT] AND “humans”[MeSH Terms]).

## CQ6-4: What nutritional support is recommended for AKI treatment?

*Recommendation*: We suggest that the administration of calorie and protein as nutritional support for AKI treatment be tailored to the severity and the underlying disease. For severe AKI, we recommend enteral nutrition whenever possible. Unless there is an advanced electrolyte imbalance, strict protein restriction is not necessary.

*Strength of recommendation*: 2

*Quality of evidence*: D

### Summary of evidence

Since the publication of the KDIGO guideline, there have been no RCTs regarding nutritional support with subjects limited to AKI patients. The KDIGO guideline recommends a calorie intake of 20–30 kcal/kg/day for AKI patients of any stage. The desired amounts of protein administration are 0.8–1.0 g/kg/day in hypermetabolic AKI patients who do not require dialysis and 1.7 g/kg/day in hypermetabolic patients undergoing CRRT; when possible, nutrition through the enteral route is preferred.

### Commentary

Acute kidney injury (AKI) is the form of organ failure to which severely ill patients are most susceptible. Thus, the metabolism is greatly affected by the primary disease, the malnutrition severity, the presence of comorbid organ failure, and the performance of renal replacement therapy (RRT). Therefore, the target levels of the calorie intake and the necessary protein should ideally be tailored to the individual pathology; however, the specific efficacy of nutritional support for AKI has not been demonstrated [194, 195].

Enteral nutrition is considered to be more effective than intravenous nutrition for intestinal mucosa maintenance, bacterial translocation, and the prevention of organ dysfunction. In the meta-analyses of studies involving critically ill patients (including AKI patients), the initiation of enteral nutrition within 24 h after intensive care unit (ICU) admission was shown to significantly reduce the mortality and the incidence of infectious complications and to shorten the hospital stay lengths; however, negative results have also been reported [196–199]. In order to provide sufficient calorie, amino acids, and protein, a combination of intravenous and enteral nutrition is sometimes considered. A group of patients who only received vitamins and trace elements through enteral nutrition for the first 7 days following ICU admission and started intravenous nutrition on day 8 (late-initiation group) demonstrated a significant increase in early discharge (alive) from the ICU/hospital, as well as reductions in the incidence of infections, the number of patients on mechanical ventilation for > 2 days, the duration of RRT, and the health care costs [200].

The target calorie provision levels can be determined with a simple body mass conversion equation (25 kcal/kg/day), a calorie consumption prediction equation (Harris-Benedict equation), or the measurement of the energy consumption with an indirect calorimeter. In the first 7 days of sepsis treatment of patients who are not yet critically ill and are not malnourished, energy replenishment by enteral nutrition is recommended; however, supplemental intravenous nutrition to reach the target energy level is not recommended, as it can affect the prognosis adversely [201]. The preferable method to reach the target energy level is to start with a small amount of energy and to increase it gradually based on factors such as the presence of aspiration and/or regurgitation of gastric contents and diarrhea. In obese patients, it must be noted that the use of the actual body weight in the prediction formula will cause an overestimation of the target energy provision level. Based on the reports that intensive insulin therapy is not useful for mortality reduction, the initiation of insulin control at a blood glucose level ≥ 180 mg/dL and the setting of a target blood glucose level of 144–180 mg/dL can be considered valid in severe AKI [3, 202, 203]. At the same time, AKI is reported to occur frequently in acute myocardial infarction patients with a blood glucose level ≥ 200 mg/dL at hospital admission [204, 205].

Protein restriction for the prevention or delay of RRT initiation is not recommended; however, it can be considered when an advanced electrolyte imbalance is present. In non-hypermetabolic AKI patients who do not require RRT, a protein provision of 0.8–1.0 g/kg/day is recommended. A protein provision of < 1 g/kg/day can induce negative nitrogen balance in patients undergoing RRT, due to the factors such as the loss of approximately 10–15 g/day of amino acids, especially in continuous renal replacement therapy (CRRT). Therefore, in hypermetabolic patients undergoing CRRT, the KDIGO guideline recommends the administration of 1.7 g/kg/day of protein to account for the amount of protein loss, while a protein intake of 2.5 g/kg/day is reportedly needed to achieve a positive nitrogen balance [206, 207]. However, the excess provision of amino acids is indicated to potentially cause azotemia and prolonged RRT [208]. During CRRT, commercially available dialysates and replacement fluids in Japan can cause hypokalemia and hypophosphatemia, and the latter is reported to delay weaning from mechanical ventilation; therefore, appropriate supplementation of potassium and/or phosphate through intravenous or enteral nutrition can be beneficial [194, 209–211]. Outside of Japan, a CRRT dialysate containing 4.0 mEq/L potassium and 3.7 mg/dL phosphorus has been developed [212]. However, a switch from CRRT to intermittent renal replacement therapy (IRRT) can easily cause an electrolyte imbalance, thereby calling for a reexamination of the content of the intravenous nutrition or enteral nutrition, including the total fluid volume; in particular, the risk of hyperkalemia must be kept in mind.

There is no clear evidence to recommend nutritional support for mild AKI without fluid overload, dehydration, or an electrolyte imbalance. The International Nutrition Survey conducted a recent international cross-sectional study on nutritional intervention, in which nine Japanese facilities participated. In this study, the calorie sufficiency rate, protein sufficiency rate, and nutrition provision rate, and in nearly all other parameters in Japanese ICU patients were found to be below the global mean; in addition, the initiation of enteral nutrition was demonstrated to be late. Further research is needed.

### Literature review

PubMed was searched for relevant studies published between January 2012 and April 2016, and papers related to the present CQ were abstracted from the search results. The literature published before January 2012 was referenced from the KDIGO Clinical Practice Guideline for Acute Kidney Injury.

## CQ7-1: Should blood purification for AKI be initiated early?

*Recommendation*: There is little evidence to support the idea that the early initiation of blood purification for AKI improves the outcomes. The timing of the initiation should be decided in broad consideration of the clinical symptoms and disease conditions.

*Strength of recommendation*: Not graded

*Quality of evidence*: C

### Summary of evidence

Out of nine relevant RCTs, three were performed at a single center (two involving patients having undergone cardiac surgery, one involving ICU patients); in all three RCTs, the early initiation of blood purification was associated with a reduced mortality. However, in a meta-analysis that included multicenter RCTs, the efficacy of the early initiation of blood purification was not supported.

### Commentary

There has been a consensus that emergent blood purification should be initiated for serious, life-threatening disease states. The KDIGO Clinical Practice Guideline for Acute Kidney Injury (AKI) also states that renal replacement therapy (RRT) should be initiated immediately in the event of potentially fatal changes in the body fluid, electrolyte, and acid-base balance (not graded). The indications for emergent RRT in clinical settings are listed in Table 11.

**Table 11:** Indications for emergent renal replacement therapy

Fluid overload resistant to diuretics	
Hyperkalemia or rapid elevation of serum potassium	
Uremic symptoms (pericarditis, consciousness disturbance with unknown etiology)	
Severe metabolic acidosis	

In observational studies from the 1960s that examined the timing of blood purification initiation, hemodialysis was demonstrated to improve the survival rates not when initiated after AKI had progressed to the point at which symptoms of uremia were present, but when initiated before that point. Since 2000, multiple observational studies have examined the initiation of blood purification when the blood urea nitrogen (BUN) is at a level even lower than that set in the aforementioned observational study. Bagshaw et al. conducted a meta-analysis of 15 clinical studies (including two randomized controlled trials [RCTs], 4 prospective observational studies, and 9 retrospective observational studies) [213]. Although the early initiation of blood purification was found to be associated with favorable outcomes, the timing of the initiation across the different studies examined was significantly heterogeneous; therefore, the early initiation of blood purification could not be definitively recommended.

The effect of the early initiation of blood purification on death has been examined in nine RCTs to date—including some that were not covered in Bagshaw et al.’s meta-analysis. Bouman et al. randomly assigned intensive care unit (ICU) patients presenting with oliguria to early high-volume, early low-volume, and late low-volume hemofiltration, with the survival at day 28 and the recovery of the renal function as primary endpoints; however, the three groups did not demonstrate any differences in their survival at day 28 or their recovery of the renal function [214]. An Indian RCT involving patients who developed AKI in the hospital divided the subjects into two groups: a group in which dialysis was initiated early, when the BUN was ≥ 70 mg/dL or the serum creatinine (sCr) was ≥ 7 mg/dL (*n* = 102), and a control group in which dialysis was initiated upon fluid overload, hyperkalemia, or any other indication for emergent dialysis (ultimately, BUN 100.9 ± 32.6 mg/dL, sCr 10.41 ± 3.3 mg/dL; *n* = 106). The comparisons of these two groups revealed no significant differences in the mortality or the recovery of the renal function [215]. In a Canadian open-label pilot trial [216] reported in 2015, patients with volume replete AKI were randomly assigned to an early treatment group (*n* = 48) or a standard treatment group (*n* = 52); the mortality and the recovery of the renal function did not show any significant differences between the two groups. In two single-center RCTs involving post-cardiac surgery patients [217, 218], the early initiation of blood purification was associated with a reduced mortality. In a multicenter RCT (the HEROICS study) [219], patients experiencing shock requiring catecholamine support following cardiac surgery were randomly assigned to one of the two groups: an early hemofiltration group (80 mL/kg/h for 48 h) or a standard therapy group that included continuous hemodiafiltration (CHDF) if necessary (*n* = 112 for both groups). The two groups did not demonstrate a significant difference in their mortality or recovery of the renal function.

Two additional RCTs involving AKI patients in the ICU were reported in May 2016. In a French multicenter RCT (the AKIKI trial) [220], critically ill patients with severe AKI (stage 3) who required mechanical ventilation or catecholamine infusion were randomly assigned to one of the two groups: an early initiation group (*n* = 311), in which RRT was initiated immediately after randomization, or a delayed initiation group (*n* = 308), in which RRT was not initiated until a criterion such as hyperkalemia, metabolic acidosis, pulmonary edema, a high BUN level (> 112 mg/dL), or oliguria (> 72 h) was met. The investigation of the utility of early RRT did not reveal a significant difference in the 60-day mortality of the two groups. In a German single-center RCT (the ELAIN trial) [221], 231 critically ill patients with stage 2 AKI and a plasma NGAL > 150 ng/mL were randomly assigned to an early group (initiation of RRT immediately after randomization) or a delayed group (initiation of RRT upon progression of AKI to stage 3 or the presence of any absolute indications); the assessment of the 90-day mortality found the latter to be significantly reduced in the early group. However, in the AKIKI trial, the RRT initiation in the early group was late, upon validation of stage 3 AKI; this timing corresponded to the delayed group in the ELAIN trial. Moreover, in the ELAIN trial, RRT was initiated as continuous renal replacement therapy (CRRT) and was performed for at least 1 week in all patients; however, in the AKIKI trial, CRRT was performed as the sole method of RRT in only 30% of patients. Although both of these RCTs examined the early initiation of RRT, it must be noted that they differed in their timing of initiation and their treatment modalities.

**Fig. 5:**
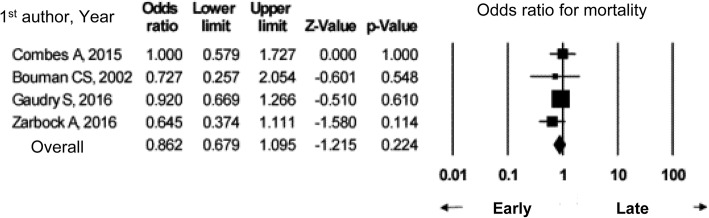
Meta-analysis for early initiation of blood purification (28- or 30-day mortality)

Among the nine RCTs reported to date, those that recorded the 28-day or 30-day mortality were subjected to a meta-analysis; this meta-analysis did not support the efficacy of early initiation (Fig. 5). Moreover, a meta-analysis that included the AKIKI [220] and ELAIN [221] trials was published [222]. In this analysis of six RCTs, including the AKIKI and ELAIN trials as well as the three previously mentioned RCTs involving non-post-cardiac surgery patients [214–216], the early initiation of blood purification did not show evident efficacy in terms of either mortality (relative risk 0.93, 95% confidence interval 0.68–1.26) or recovery of the renal function (relative risk 0.88, 95% confidence interval 0.48–1.62).

As of August 2016, the multicenter STARRT RCT [223] is ongoing. In France, a multicenter RCT assessing the utility of early initiation of RRT for septic AKI (corresponding to KDIGO stage 3) is ongoing (the IDEAL-ICU study [224]). Both of these RCTs are larger in scale than the previous investigations; therefore, the results obtained may lead to new directions about early initiation.

### Literature review

PubMed was searched for relevant studies published up to August 2015, and papers related to the present CQ were identified from the search results. Important manuscripts published after the search period ([220, 222, 221]) were also incorporated into our recommendation.

## CQ7-2: What indicators should be used for discontinuation of the blood purification for AKI?

*Recommendation*: Improvements in the clinical data and the urine output can be used to determine the timing of the blood purification discontinuation.

*Strength of recommendation*: Not graded

*Quality of evidence*: C

### Summary of evidence

There have been no RCTs relevant to the discontinuation of blood purification. In three observational studies, the urine output and SOFA score were reported to be predictors of the possibility to wean patients from blood purification.

### Commentary

The blood purification for acute kidney injury (AKI) is stopped when the renal function sufficiently recovers. However, very few studies have examined the criteria for discontinuation of the blood purification for AKI. Our literature search found three observational studies that identified predictors of the possibility to wean patients from blood purification to date. Wu et al. retrospectively examined AKI patients (*n* = 304) who required renal replacement therapy (RRT) in the intensive care unit (ICU) after surgery [225]. Of the 94 patients in whom RRT was discontinued, 30 patients needed to resume it within 30 days; the duration of the RRT (odds ratio [OR] 1.06, 95% confidence interval [CI] 1.02–1.10), the SOFA score at cessation (OR 1.44, 95% CI 1.13–1.83), oliguria (< 100 mL over 8 h) (OR 4.17, 95% CI 1.07–16.13), and an age over 65 years (OR 6.35, 95% CI 1.61–24.99) were identified as predictors of discontinuation failure. Kawarazaki et al. retrospectively examined AKI patients (*n* = 343) in Japanese ICUs who required continuous renal replacement therapy (CRRT) [226]. The comparison of an early recovery group (those who could discontinue CRRT within 48 h of initiation; *n* = 52) and a control group that excepted patients who died early (*n* = 239) revealed that the urine output upon CRRT initiation (mL/h) (OR 1.02, 95% CI 1.01–1.03), the SOFA score upon CRRT initiation (OR 0.87, 95% CI 0.78–0.96), and the time from ICU admission to CRRT initiation (in days) (OR 0.65, 95% CI 0.43–0.87) were significantly associated with early weaning from CRRT. It is of note that the urine output and SOFA score data were collected upon CRRT initiation, and some patients who ceased the CRRT within 48 h may not have required blood purification; therefore, these results should be interpreted with caution.

The urine output is perhaps the most clinically emphasized predictor; one useful reference is a sub-analysis of the BEST study (*n* = 1006), in which Uchino et al. examined AKI patients in ICUs in 23 countries [227]. Those who did not require RRT for at least 7 days after the initial discontinuation were defined as the success group (*n* = 313), while those who had to resume RRT within 7 days after the initial discontinuation were defined as the repeat-RRT group (*n* = 216). Comparisons of the two groups revealed that the urine output was the most useful predictor of RRT weaning; the cutoff values for the use and the non-use of diuretics were 2330 mL/day (approx. 100 mL/h) and 436 mL/day (approx. 20 mL/h), respectively.

In the previously cited sub-analysis of the BEST study, the serum creatinine (sCr) was also reported to be a significant predictor of weaning (OR 0.996, 95% CI 0.994–0.998). Creatinine is produced from creatine in the muscle tissue and is released into the blood. Both blood purification and the kidneys of the patients remove creatinine. The balance of creatinine between muscle production and elimination by blood purification and kidneys defines the sCr. Therefore, if the sCr level remains constant for at least 2–3 days, the production and elimination can be considered equal. The phenomenon by which the amount of sCr remains constant for several days before suddenly decreasing sharply without changing blood purification doses—called “spontaneous fall”—indicates that the renal function has recovered. In the VA/NIH ATN study [228] that examined the association between the dialysis doses and the AKI outcomes, the recovery of the renal function was defined as a urine output > 30 mL/h in 6 h of collection or a spontaneous fall in the sCr. The following protocol was adopted: if the creatinine clearance in the 6-h urine collection was > 20 mL/min, the CRRT was discontinued; if the creatinine clearance was < 12 mL/min, the CRRT was continued; if the creatinine clearance was between 12 and 20 mL/min, the decision to continue or discontinue the CRRT was left to the clinician.

However, in AKI and advanced chronic kidney disease (CKD), creatinine not only undergoes glomerular filtration but is also excreted into the urine due to re-secretion from the renal tubule; consequently, the creatinine clearance is greater than the actual glomerular filtration rate (GFR). In addition, if the sCr continues to decrease in 6-h urine collection, the sCr value selected for use in the GFR calculation may result in an overestimation or underestimation of the GFR. At the AKI recovery stage, in which the renal function fluctuates dynamically, the sCr and creatinine clearance are markedly unreliable; however, due to the absence of other appropriate endpoints, the sCr may be an acceptable basis upon which to determine whether to discontinue the blood purification, with the above-described background considered.

### Literature review

PubMed was searched for relevant studies published up to August 2015, and papers related to the present CQ were identified from the search results.

## CQ7-3: How should the blood purification dose be determined for AKI?

*Recommendation*: There is no evidence allowing for the recommendation of an optimal blood purification dose. The dose must be determined individually by considering disease conditions.

*Strength of recommendation*: 2

*Quality of evidence*: B

### Summary of evidence

Increasing the dose of blood purification for AKI to higher than the level recommended as the international standard (20–25 mL/kg/h) has not been reported to improve the AKI outcomes. No RCTs have compared the blood purification dose covered by health insurance in Japan (10–15 mL/kg/h) to that recommended internationally; only two observational studies have evaluated this issue, and neither observed a significant difference in mortality. Thus, there is no definitive evidence to support the need to change the dose used in Japan to that recommended as the international standard.

### Commentary

The appropriate dose of renal replacement therapy (RRT) for acute kidney injury (AKI) has been investigated so far. Several studies have reported that increased doses do not lead to improved outcomes [214, 228–230]; in addition, there is insufficient evidence to determine an optimal dose.

The dose of continuous renal replacement therapy (CRRT) for AKI was firstly examined by a 2000 study by Ronco et al. [231]. A total of 425 AKI patients who required continuous hemofiltration (CHF) were randomly assigned a filtration flow rate (QF) of 20, 35, or 45 mL/kg/h. The comparisons of the three groups revealed respective survival rates of 41, 57, and 58%; the 20 mL/kg/h group demonstrated a significantly lower survival rate than the other two groups, while there was no significant difference between the 35 mL/kg/h group and the 45 mL/kg/h group. Since then, two multicenter, large-scale randomized controlled trials (RCTs) have been reported (in 2008 and 2009, respectively) [228, 230]; unlike in the trial reported by Ronco et al., these two RCTs found that increased doses of RRT for AKI did not improve the outcomes. In the ATN study [228], 1124 AKI patients who required RRT were randomly assigned to a standard therapy group or an intensive therapy group, and the two groups’ mortality and recovery of the renal function were compared. In the standard therapy group, hemodynamically stable patients underwent hemodialysis (HD) three times a week, while hemodynamically unstable patients either underwent continuous hemodiafiltration (CHDF) at a rate of 25 mL/kg/h or received sustained low-efficiency dialysis (SLED) three times a week. In the intensive therapy group, hemodynamically stable patients underwent HD six times a week, while hemodynamically unstable patients either underwent CHDF at a rate of 35 mL/kg/h or received SLED six times a week. The comparisons revealed no significant differences in the mortality or recovery of the renal function of the two groups. In the RENAL study [230], 1508 AKI patients were randomly assigned to an intensive therapy group (35 mL/kg/h CHDF) or a standard therapy group (25 mL/kg/h CHDF), and the two groups’ mortality and recovery of the renal function were compared. Likewise, the two groups in this study did not demonstrate any significant differences in their mortality or recovery of the renal function. Based on the results of two multicenter, large-scale RCTs, the recent KDIGO Clinical Practice Guideline for AKI [3] recommends a CRRT dose of 20–25 mL/kg/h. However, both the ATN and RENAL studies examined AKI collectively with a wide variety of causes, including ischemia, nephrotoxic substances, and sepsis; to date, there have been very few studies of the optimal doses in function of the underlying disease. Regarding septic AKI, four RCTs [232–235] have compared the CRRT outcomes from doses of 35–45 mL/kg/h and larger doses (65–100 mL/kg/h); in these RCTs, increased doses were not found to improve the outcomes. Based on the above, there is currently no definitive evidence allowing for the recommendation of optimal doses according to the underlying disease. However, in the event of acute hyperkalemia—such as in tumor lysis syndrome—the blood purification doses must be temporarily increased or otherwise tailored to the individual pathologies.

The common dose of CRRT for AKI in Japan (10–15 mL/kg/h) is generally smaller than the recommended dose outside of Japan (20–25 mL/kg/h). This seems to be because Japanese health insurance only covers a dialysis dose of approximately 15 L/day. No RCTs have compared the standard Japanese dialysis dose of 10–15 mL/kg/h with the recommended international dose of 20–25 mL/kg/h; however, two retrospective observational studies [236, 237] have concluded that the standard Japanese dose did not lead to worse outcomes. As mentioned above, although Ronco et al. reported an optimal blood purification dose of 35 mL/kg/h, this was controverted by two subsequent large-scale RCTs. However, there is currently insufficient evidence to determine that 20–25 mL/kg/h is an optimal dose, and the standard Japanese dose must be examined as well. Moreover, while it is unknown whether the reduction of doses to below the standard Japanese dose of 10–15 mL/kg/h would worsen the outcomes, we would like to address that there is no evidence to recommend the reduction of dialysis doses.

The appropriate doses of HD for AKI have been examined in three RCTs [214, 228, 238]. In those, the different groups demonstrated no significant differences in their mortality or recovery of the renal function. For intermittent renal replacement therapy (IRRT) or extended RRT, the KDIGO Clinical Practice Guideline for AKI recommends a weekly standardized dialysis dose (Kt/V) of 3.9. However, there is insufficient evidence to establish this as the optimal hemofiltration dose for AKI. An RCT that compared HD and predilution online HF rather than HD doses was reported in 2012 [239]. The mean volume of infusate in predilution online HF was 81 L; the HF and HD groups did not demonstrate significant differences in their mortality or recovery of the renal function. In a prospective study by Schiffl et al. that compared a daily HD group with an alternate-day HD group [238], the daily HD group demonstrated a significantly lower mortality and a significantly earlier recovery of the renal function. However, several issues have been raised with this study: the HD doses were extremely low, and the randomization was inadequate. The Hannover Dialysis Outcomes Study randomly assigned 156 AKI patients to a standard dialysis group and an intensified dialysis group and compared the mortality and the recovery of the renal function in the two groups [240]. The standard dialysis was dosed to maintain a blood urea nitrogen (BUN) level of 120–150 mg/dL, while the intensified dialysis was dosed to achieve a target BUN level of < 90 mg/dL. However, the two groups did not demonstrate significant differences in their mortality or recovery of the renal function.

### Literature review

PubMed was searched for relevant studies published up to July 2015, and papers related to the present CQ were identified from the search results.

## CQ7-4: Should blood purification for AKI be performed continuously or intermittently?

*Recommendation*: In hemodynamically stable patients, blood purification may be performed either continuously or intermittently. In hemodynamically unstable patients, continuous blood purification is preferable.

*Hemodynamically stable patients*:

*Strength of recommendation*: 2

*Quality of evidence*: B

*Hemodynamically unstable patients*:

*Strength of recommendation*: Not graded

*Quality of evidence*: C

### Summary of evidence

Several RCTs and meta-analyses have compared CRRT with IRRT, but none have demonstrated differences in mortality. It must be noted that some of these RCTs excluded hemodynamically unstable patients and that none limited their subjects to hemodynamically unstable patients. In meta-analyses of sustained low-efficiency dialysis (SLED), which combines the advantages of both CRRT and IRRT, comparisons with CRRT revealed no significant difference in the mortality.

### Commentary

The optimal modality of blood purification for acute kidney injury (AKI) has been investigated. The primary continuous and intermittent modalities used in Japan are continuous hemodiafiltration (CHDF) and hemodialysis (HD), respectively [241]. The choice of therapy is generally based on a consideration of various factors, including the patient’s hemodynamics and anticoagulation, the facility’s equipment, and the staff’s experience and manpower. The advantages and disadvantages of these modalities are summarized in Table 12. The greatest advantage of continuous renal replacement therapy (CRRT), which is mostly conducted with CHDF in Japan, is that its effects on the hemodynamics are minimized as it removes the body fluids and solutes gradually. CRRT is also associated with a reduced risk of cerebral edema [242]. However, continuous blood purification not only restrains the patient over a long period of time but also places a great burden on the medical care staff. In addition, the continuous administration of anticoagulants increases the risk of hemorrhage. Intermittent renal replacement therapy (IRRT), which is mostly conducted with HD in Japan, removes the body fluids and solutes quickly, thereby easily affecting the hemodynamics and increasing the risk of cerebral edema. However, in addition to being completed faster, IRRT places a lesser burden on the staff and poses a lower risk of hemorrhage than CRRT. As CRRT and IRRT possess evidently different characteristics, direct comparisons of their utility have been found to be worthless [243].

**Table 12:** Comparison between IRRT and CRRT

	Advantage	Disadvantage
IRRT	Rapid correction of fluid and electrolyte	Hemodynamic intolerance
	Patient’s mobility	Rebound phenomenon
	Anticoagulation/bleeding disorders	
	Low cost	
CRRT	Hemodynamic tolerance	Requirement of continuous anticoagulation
	No osmotic cellular shift	High cost
		Workload for medical staff

For the present CQ, we abstracted a total of 15 randomized controlled trials (RCTs) [244–258] and 8 meta-analyses [259–266] that compared the utility of the two modalities of renal replacement therapy (RRT) for AKI. A primary meta-analysis was reported as part of a Cochrane joint project in 2008 [265]. An analysis of 15 RCTs involving a total of 1550 AKI patients who required RRT revealed no significant differences between CRRT and IRRT in the in-hospital mortality, the ICU mortality, or the discontinuation of RRT in surviving patients. Several other meta-analyses have demonstrated similar results [260–263]. However, in a meta-analysis of 13 studies (including three RCTs) by Kellum et al., while CRRT and IRRT yielded no significant difference in mortality, adjustments for the severity of the illness and the study quality revealed that the risk of death was significantly reduced in CRRT (relative risk [RR] 0.72, 95% confidence interval [CI] 0.60–0.87) [259]. In Schneider et al.’s meta-analysis of 23 studies (including 7 RCTs) related to the rates of dialysis dependence, although the risk of dialysis dependence was significantly higher in patients undergoing IRRT (RR 1.73, 95% CI 1.35–2.20), there was no significant difference when the analysis was limited to RCTs [264]. The KDIGO guideline and the Surviving Sepsis Campaign Guideline (SSCG) 2012 [267] considered the same matter as the present CQ and gave similar recommendations based on the results described above.

It must be noted that two RCTs [245, 254] excluded hemodynamically unstable patients. Theoretically and empirically, CRRT has been considered useful, and has therefore been used, for the treatment of hemodynamically unstable patients. No RCT has ever compared CRRT and IRRT in hemodynamically unstable patients. Based on the above, our expert opinion is that CRRT is preferable for hemodynamically unstable patients. However, there is no standard opinion on the degree of instability at which CRRT should be chosen. In addition, many hemodynamically unstable patients are of course critically ill and may have comorbid coagulation disorders, such as disseminated intravascular coagulation (DIC). HD may be more useful in patients with an advanced bleeding tendency, as it can be performed with only a short course of anticoagulant administration. In addition, the selection of a modality must take into account the equipment at the facility, the staff’s experience and resources, and a variety of other factors unrelated to the patient. The modality should be decided by a physician with sufficient knowledge and experience of blood purification (i.e., an intensive care specialist, nephrologist, or dialysis physician) according to the patient’s disease condition.

Sustained low-efficiency dialysis (SLED), or extended daily dialysis (EDD), uses the lower blood flow and the lower dialysate flow and is performed more frequently than standard HD; it incorporates the advantages of both CRRT and IRRT and is now performed widely. Although no RCTs have compared SLED and intermittent hemodialysis (IHD), the effects of SLED on the hemodynamics are reported to be the same as those of CRRT [268]. Recently, Zhang et al. conducted a meta-analysis of 17 studies (including 7 RCTs) that compared EDD and CRRT for AKI [266]. In the RCTs, no significant difference was observed in the mortality of the different groups; however, in the ten observational studies, the risk of death was lower with EDD (RR 0.86, 95% CI 0.74–1.00). No significant difference in the recovery of the renal function was evidenced by the RCTs or the observational studies. By establishing appropriate implementation conditions, not only CRRT but also EDD may be performed in hemodynamically unstable patients.

### Literature review

PubMed was searched for relevant studies published up to July 2015, and papers related to the present CQ were identified from the search results.

## CQ7-5: Should nafamostat mesilate be used as an anticoagulant in blood purification for AKI?

*Recommendation*: Nafamostat mesilate may be considered for patients with a high risk of bleeding. For patients with active bleeding, blood purification without the use of anticoagulants may also be considered.

*Strength of recommendation*: Not graded

*Quality of evidence*: C

### Summary of evidence

There have only been two RCTs involving the use of nafamostat mesilate as an anticoagulant during blood purification for AKI (nafamostat mesilate vs no anticoagulant). No significant difference was observed in the survival outcomes. Two observational studies that compared the use of nafamostat mesilate and heparin also found no significant difference in the survival outcomes.

### Commentary

The blood coagulates when it comes into contact with anything including artificial materials other than vascular endothelial cells. Therefore, renal replacement therapy (RRT)—which involves extracorporeal circulation—usually requires the use of anticoagulants. Patients with severe acute kidney injury (AKI) requiring RRT frequently present with a hemorrhagic complication. Thus, the use of anticoagulants that pose the lowest possible risk of bleeding is required. Japanese health insurance currently covers four types of anticoagulants for RRT: unfractionated heparin, low molecular weight heparin (LMWH), nafamostat mesilate (NM), and argatroban. Citrate is widely used as an anticoagulant outside of Japan. Although it can be used in Japan as well, this is not frequent, since its use as an anticoagulant for RRT is considered off-label. In the BEST kidney study [34], which included an examination of the anticoagulants used in continuous renal replacement therapy (CRRT) for AKI, the most commonly used anticoagulant was unfractionated heparin (42.9% of patients), followed by no anticoagulant (33.1%), citrate (9.9%), NM (6.1%), and LWMH (4.4%).

When possible, CRRT without anticoagulation is the safest option for AKI patients, as it does not increase the risk of bleeding; however, the lifespans of the filter and circuit may be shortened, and CRRT cannot be performed without anticoagulation in all patients. In Japan, NM is widely used as an anticoagulant in CRRT for AKI since it has a short half-life and is associated with a relatively lower risk of bleeding than other anticoagulants. However, it has not been approved only in the limited countries. In addition, NM causes adverse effects, including agranulocytosis, hyperkalemia, and anaphylactoid reactions [269–272]. In Japan, unfractionated heparin is the most commonly used anticoagulant for hemodialysis (HD) in chronic dialysis patients. However, due to concerns over the risk of bleeding, it is seldom used as an anticoagulant in CRRT for AKI in Japan. Similarly, although LMWH is also associated with a lower risk of bleeding than unfractionated heparin, the test that indicates anticoagulant action (the anti-Xa assay) is not common. Therefore, LMWH is infrequently used as an anticoagulant in CRRT for AKI [34].

Five studies have examined the use of NM as an anticoagulant in CRRT for AKI [34, 273–276]. Only two of these studies were randomized controlled trials (RCTs) [273, 274], while one was a prospective observational study [34] and two were retrospective studies [275, 276]. Both of the RCTs compared NM with no coagulation, and neither observed a significant difference in the survival outcomes of the groups. In one RCT, the lifetime of the hemofilter was found to be significantly longer with NM than without coagulation. The two groups demonstrated no significant difference in their hemorrhagic complications. A prospective observational study (the BEST kidney study) and a retrospective study by Hwang et al. [276] also observed no significant differences in the survival outcomes. In a retrospective observational study in which continuous hemodiafiltration (CHDF) was performed without anticoagulation in patients at a high risk of bleeding, Baek et al. reported that only NM was used when the hemofilter lifespan was less than 12 h [275]. The in-hospital mortality was significantly lower in the NM group (anticoagulation-free group vs NM group: 64.6 vs 41.9%, *p* = 0.003), while there was no significant difference between the groups in the transfusion volume (anticoagulation-free group vs NM group: 0.7 vs 0.7 units/day). In addition to the five studies cited above, another study in which NM was compared with heparin was recently published in Japan [277]. Despite being a retrospective observational study, its analysis featured propensity score-matched cohorts. Although the mortality was not examined, hemorrhagic complications were significantly less frequent in the NM group, while there was no significant difference in the filter lifespan.

Citrate is commonly used as an anticoagulant in CRRT for AKI outside of Japan. Although the safety and efficacy of citrate have been assessed, its use as an anticoagulant in CRRT for AKI is considered off-label in Japan. Although no RCTs have compared citrate with NM, ten RCTs have compared citrate and unfractionated heparin [278–287]; in the six RCTs that examined the survival outcomes [279, 282–284, 286, 287], no significant difference was observed. Eight studies found the filter lifespan to be significantly longer with citrate [278, 279, 282–287], while two observed no significant difference [280, 281]. The frequency of hemorrhagic complications with citrate and unfractionated heparin was found to be either equal or significantly lower with citrate. Five more RCTs examined the use of LMWH as an anticoagulant in CRRT [288–292]; three of them [288, 289, 291] compared LMWH with unfractionated heparin, while two [290, 292] compared LMWH with citrate. Only one of the five RCTs [290] examined the clinical outcomes; in this RCT, the mortality was significantly lower in the citrate group. With regard to bleeding, only one RCT found a significant difference [291]; however, the results related to the filter lifespan varied across studies.

### Literature review

PubMed was searched for relevant studies published up to December 2015, and papers related to the present CQ were identified from the search results. The study by Makino S et al. [277], which was published after the search period, was found through a hand search.

## CQ7-6: What membrane material should be chosen for blood purification in AKI?

*Recommendation*: There is no evidence for the recommendation of a specific membrane material to improve the outcomes.

*Strength of recommendation*: 2

*Quality of evidence*: C

### Summary of evidence

The majority of blood purification filters currently used in Japan are biocompatible high-flux membranes. However, no studies have found the differences in these membranes to affect the AKI outcomes or the recovery of the renal function. For AKI—and particularly septic AKI—blood purification is performed in Japan to improve hypercytokinemia by using the principle of adsorption; however, there is no high-level evidence for the effect of this blood purification method on the outcomes.

### Commentary

The blood purification membrane materials currently used in Japan include cellulose triacetate (CTA), polymethyl methacrylate (PMMA), ethylene vinyl alcohol (EVAL), polysulfone (PS), polyethersulfone (PES), polyarylethersulfone (PAES), and polyester polymer alloy (PEPA). These materials will activate complement system less than cuprophan and other regenerated celluloses that have been used since the 1960s and are considered to be highly biocompatible membranes. Many of these membranes currently in use have been developed as high-flux (HF) membranes, with the goal to remove the β2 microglobulin (β2MG) and other small molecule proteins.

The therapeutic effects of each membrane material have been compared in a few small-scale randomized clinical trials (RCTs) that primarily featured comparisons of regenerated cellulose with synthetic polymeric membranes. These RCTs have also featured comparisons of HF membranes with the low-flux (LF) membranes that were developed prior to the existence of HF membranes. With regard to the present CQ, we found seven relevant RCTs that compared different types of membranes [293–299].

Five of these RCTs primarily compared the effects of the differences in the membranes’ biocompatibility, along with the effects of the differences between LF and HF membranes. Schiff et al. [293] examined the recycled cellulose Cuprophan® (LF membrane) and the synthetic polymer polyacrylonitrile (PAN; HF membrane) in two groups of 26 postoperative acute kidney injury (AKI) patients each and compared the results of the two groups. In later trials, Jörres et al. [294] compared 76 AKI patients in whom Cuprophan® (LF membrane) was used with 84 patients in whom PMMA (LF membrane) was used. Meanwhile, Gastaldello et al. [295] and Albright et al. [296] compared cellulose acetate (LF membrane) with PS (HF membrane)—both of which are improved versions of Cuprophan®—in AKI patients. However, none of the above three trials observed differences in the outcomes or in the recovery of the renal function. In 2008, Cochrane reported a meta-analysis of 1100 patients from the five RCTs cited above and from five others (ten RCTs in all); the comparisons of the biocompatible membranes (synthetic polymeric membranes, *n* = 575) with the bioincompatible membranes (regenerated cellulose membranes, *n* = 525) revealed no significant differences in the mortality (relative risk 0.93, 95% confidence interval 0.81–1.07) or in the recovery of the renal function (*n* = 1038, relative risk 1.09, 95% confidence interval 0.90–1.31) [300]. Although these results are not directly relevant to comparisons of the synthetic polymeric membranes mainly used today, it should be acknowledged that no significant differences have been found between synthetic polymeric membranes and the so-called bioincompatible regenerated cellulose membranes.

Jones et al. [297] compared the survival rates for the use of the two synthetic polymeric membranes PAN (*n* = 97) and PS (*n* = 100) (both HF membranes) in the continuous hemodialysis (CHD) of ventilated patients with AKI. No significant difference was observed (PAN 29%, PS 27%). In the 2000s, another trial compared HF and LF membranes made of the same material. Ponkivar et al. [298] compared HF membranes (*n* = 34) and LF membranes (*n* = 38) both made of PS in AKI patients; the two groups’ results were similar. Based on the above, none of the blood purification membranes currently used in Japan can produce better AKI treatment outcomes.

The core of the pathophysiology of septic AKI is assumed hypercytokinemia. The improvement of hypercytokinemia may be useful for the improvement of the performance status and of AKI. This has led to attempts at blood purification designed to remove all types of cytokines. Haase et al. [299] conducted a crossover RCT in which ten sepsis patients with AKI classified by the RIFLE criteria as Failure underwent hemodialysis (HF) using a standard HF membrane (with in vivo molecular weight cutoff values of 15–20 kD) and a membrane with a larger pore size (50–60 kD). Comparisons of the cytokine removal efficiency revealed that the use of a membrane with a larger pore size significantly reduced the blood concentrations of the cytokines IL-6, IL-8, and IL-10 at 4 h of HD. In Japan, PMMA membranes [301] and AN69ST membranes [302], which are based on the principle of adsorption and are considered to highly efficient at removing cytokines, have been used in attempts at blood purification. Since 2014, AN69ST membranes have been covered by health insurance in Japan for patients with severe sepsis and septic shock. However, there is no high-level evidence of the clinical efficacy of membranes with large pore sizes or of adsorption membranes. Therefore, in regard to blood purification for the treatment of sepsis, the KDIGO guideline states, “Until further evidence becomes available, the use of RRT to treat sepsis should be considered experimental.” The collection of further evidence is anticipated.

### Literature review

PubMed was searched for relevant studies published up to July 2015, and papers related to the present CQ were identified from the search results.

## CQ8: Do AKI patients require long-term follow-up?

*Recommendation*: The long-term outcomes of AKI are poor. Therefore, we suggest confirming patients’ condition at approximately 3 months later and conducting long-term follow-up in accordance with their condition.

*Strength of recommendation*: 2

*Quality of evidence*: C

### Summary of evidence

At present, there have been no RCTs to examine the long-term outcomes of AKI (≥ 12 months following onset). However, there have been systematic reviews and meta-analyses of observational studies related to the survival outcomes, cerebrovascular and cardiovascular outcomes, and renal outcomes; the most reliable and most recent one is a study by Sawhney et al. Based on the search query used in that systematic review, we further searched the literature for studies related to the survival outcomes, the cardiovascular and cerebral disease outcomes, and the renal outcomes with an extended search period. We then conducted a meta-analysis of the search results, along with a consideration of any new studies related to the survival outcomes and renal outcomes, and of observational studies related to the cerebral and cardiovascular disease outcomes. In the results of our meta-analysis, the long-term outcomes of AKI patients were consistently poor. Moreover, although there have been no meta-analyses of the long-term QOL, some observational studies report that the onset of AKI is associated with a reduced long-term QOL.

### Commentary

The term “acute renal failure” (ARF) was used for the first time in writing by Heberden et al. in 1802 [303]. Although ARF was once considered to be reversible and therefore to have a favorable outcome, Hishida et al. reported that ARF patients have extremely poor survival outcomes and cited multiple organ failure as a crucial underlying factor [304]. There were already multiple definitions of ARF [4]. In order to avoid confusion over the definition of ARF and to define acute syndromes related to the renal function more broadly, the term “acute kidney injury” (AKI) was suggested globally.

As the concept of AKI spread worldwide, many clinical studies on AKI have been performed. These studies have shown that the survival outcomes of AKI are poor [305–308], that its long-term outcomes are also poor [309], and that the stage of AKI in the intensive care unit (ICU) is correlated with the mortality [310]; as a result, perspectives on the outcomes of AKI have been changing. In 2015, Sawhney et al. reported the results of a systematic review that assembled the results of individual studies [311]; the survival outcomes and renal outcomes 1 year after the AKI onset were both shown to be poor. However, other clinically important outcomes such as the cerebral outcomes, the cardiovascular outcomes, and the quality of life (QOL) have not been examined.

#### Survival outcomes

Sawhney et al.’s systematic review reported poor long-term post-AKI survival outcomes [311]. We could not find any subsequent clinical study that yielded different conclusions about the long-term post-AKI survival outcomes.

#### Cerebral and cardiovascular disease outcomes

Several meta-analyses of existing observational studies were reported in 2015 [312–314]. Although each of these reports used different subjects and endpoints, they consistently showed that the long-term outcomes of cerebral and cardiovascular diseases in AKI patients are poor. Of note, the subjects in these meta-analyses were limited to all post-cardiovascular surgery patients (post-aortic aneurysm repair [312], post-aortic valve implantation [313], and post-cardiopulmonary bypass [314]).

#### Renal outcomes

Sawhney et al.’s systematic review revealed that the long-term post-AKI renal outcomes are poor [311]. We could not find any subsequent manuscript that yielded different conclusions about the long-term post-AKI renal outcomes.

#### QOL

As of the end of 2015, there had been few reports and no meta-analysis results relevant to the long-term post-AKI QOL; however, an observational study by Nisula et al. used the EQ5D score [315], and another by Hofhuis et al. used the SF36 [316]. In both studies, the QOL was worse in the AKI group than in the non-AKI group. We did not find any studies on the long-term prognoses of AKI that adopted the ADL or fractures as outcomes.

Based on the above, the long-term post-AKI survival outcomes, cerebral and cardiovascular outcomes, and renal outcomes can all be considered poor. Therefore, patients who develop AKI are thought to require long-term follow-up. Moreover, we recommend conducting an initial follow-up—in which it is recommended to assess the performance status and possible complications—at 3 months in order to evaluate the possible development of chronic kidney disease (CKD). We chose this timing for two reasons: (1) according to the current diagnostic criteria, CKD is defined as a kidney injury that continues for 3 months, meaning that the renal assessment at 3 months post-AKI can be considered valid; (2) we considered consistency with the KDIGO Clinical Practice Guideline for AKI [3].

### Literature review

Based on the search query used by Sawhney et al. in their systematic review [311], we developed a search query to encompass four types of outcomes: survival outcomes, cerebral and cardiovascular outcomes, renal outcomes, and the QOL. PubMed was searched for relevant studies published between January 1, 2005, and April 30, 2015. In regard to the long-term survival outcomes and the long-term renal outcomes, we abstracted results from beyond the subject period used in existing systematic reviews. With regard to the titles and abstracts, we conducted a preliminary review and selected potentially relevant manuscripts; we then conducted a secondary review of these manuscripts (full-text assessments) to identify our final target manuscripts.

## CQ9-1: Should the KDIGO diagnostic criteria for AKI be used for children?

*Recommendation*:

Age ≥ 3 months: We suggest using the KDIGO AKI diagnostic criteria to predict the survival outcomes.

Age < 3 months: We suggest using the modified KDIGO diagnostic criteria for neonates.

*Age ≥ 3 months*:

*Strength of recommendation*: 2

*Quality of evidence*: C

*Age < 3 months*:

*Strength of recommendation*: Not graded

*Quality of evidence*: D

### Summary of evidence

#### Age ≥ 3 months

Two single-center retrospective observational studies have assessed the KDIGO diagnostic criteria in sufficiently large cohorts; these studies consistently demonstrated that the KDIGO diagnostic criteria were useful for the prediction of the mortality and of other outcomes.

#### Age < 3 months

Two review papers have examined the diagnosis of AKI in neonates and have commented on the results obtained in a total of 11 observational studies. The modified neonatal KDIGO criteria were suggested with the data of associations with the AKI onset, mortality, and neurological outcomes.

#### Commentary

The early diagnosis and treatment of acute kidney injury (AKI) are crucial to the improvement of the outcomes not only in adults but also in children. Several diagnostic criteria have been suggested for children. These have included the Pediatric RIFLE (pRIFLE) (Table 13), AKIN (Table 2), and KDIGO (Table 3) criteria. It is known that the normal serum creatinine (sCr) values change with age (Table 14) [317]. As urine collection is difficult in children, the pRIFLE classification uses a Schwartz formula-based [318] calculation of the estimated glomerular filtration rate (eGFR) [319]. Due to the differences between the Japanese and Western body constitutions and renal functions, the assessment of the GFR based on the Schwartz formula is considered unsuitable for Japanese children [320]; therefore, another equation has been proposed to estimate the GFR in Japanese children [321]. The AKIN and KDIGO classifications of AKI are based on the sCr and on the duration of oliguria/anuria (i.e., a urine output < 0.5 mL/kg/h). Hereafter, we will describe studies that have compared these multiple diagnostic criteria for pediatric AKI.

**Table 13:** pRIFLE criteria

	eGFR criteria	Urine output criteria
Risk	Decrease in eGFR ≥ 25%	UO < 0.5 mL/kg/h × 8 h
Injury	Decrease in eGFR ≥ 50%	UO < 0.5 mL/kg/h × 16 h
Failure	Decrease in eGFR ≥ 75% or eGFR < 35 mL/min/1.73 m^2^	UO < 0.3 mL/kg/h × 24 h or anuria × 12 h
Loss	Complete loss of kidney function > 4 weeks	
ESKD	End-stage renal disease (dialysis dependent > 3 months)	

*eGFR* estimated glomerular filtration rate, *sCr* serum creatinine, *ESKD* end-stage kidney disease, *UO* urine output

**Table 14:** Pediatric reference ranges of serum creatinine (mg/dL)

Age	2.5 percentile		50 percentile		97.5 percentile	
3–5 months	0.14		0.20		0.26	
6–8 months	0.14		0.22		0.31	
9–11 months	0.14		0.22		0.34	
1 year	0.16		0.23		0.32	
2 years	0.17		0.24		0.37	
3 years	0.21		0.27		0.37	
4 years	0.20		0.30		0.40	
5 years	0.25		0.34		0.45	
6 years	0.25		0.34		0.48	
7 years	0.28		0.37		0.49	
8 years	0.29		0.40		0.53	
9 years	0.34		0.41		0.51	
10 years	0.30		0.41		0.57	
11 years	0.35		0.45		0.58	
	Male	Female	Male	Female	Male	Female
12 years	0.40	0.40	0.53	0.52	0.61	0.66
13 years	0.42	0.41	0.59	0.53	0.80	0.69
14 years	0.54	0.46	0.65	0.58	0.96	0.71
15 years	0.48	0.47	0.68	0.56	0.93	0.72
16 years	0.62	0.51	0.73	0.59	0.96	0.74

Sutherland et al. compared the pRIFLE, AKIN, and KDIGO diagnostic criteria in 14,795 children aged under 18 who were hospitalized for AKI [322]. The AKIN and KDIGO classifications, which both use the sCr criteria, were almost completely in agreement; however, as the eGFR-based pRIFLE classification has a higher incidence for stage 1 than the AKIN or KDIGO classifications, a larger number of patients were diagnosed with mild AKI. In all three classifications, the mortality was higher in patients with AKI than in those without AKI; particularly in the intensive care unit (ICU), the increasing severity of AKI (according to all three classifications) was associated with increased mortality. Selewski et al. used the KDIGO classification to examine the AKI outcomes in a cohort of 2415 patients in pediatric ICUs [323]. In comparison with patients who did not develop AKI, pediatric AKI patients demonstrated a significantly increased length of mechanical ventilation, a longer ICU stay, a longer duration of hospitalization, and a higher mortality rate. In addition, the length of the ICU stay was proportional to the worsening of the KDIGO AKI stages. These two single-center retrospective observational studies involved sufficient numbers of patients to demonstrate that the KDIGO classification is useful for the diagnosis of pediatric AKI. Moreover, as the KDIGO classification does not involve an estimation of the GFR but instead allows to stage AKI based on the sCr, it can be considered superior to the pRIFLE classification. Therefore, we suggest the use of the KDIGO diagnostic criteria for pediatric AKI patients aged ≥ 3 months. However, it must be noted that the use of the AKI diagnostic criteria has not yet been specifically evaluated in Japanese children.

Neonates and children aged < 3 months possess a unique background that includes immaturity and perinatal factors; therefore, children under 3 months must be considered separately from those aged ≥ 3 months. Although there have been investigations of the AKI diagnosis, treatment, and outcomes in neonates, there used to be no definitive diagnostic criteria for neonatal AKI [324, 325]. As the use of adult diagnostic tools such as the RIFLE, AKIN, and KDIGO criteria spread, their use for the diagnosis of AKI in neonates came to be researched too. Although the pRIFLE classification [318, 319] was proposed for pediatric use, it requires calculation of the eGFR and is therefore unsuitable for neonates, in whom the eGFR cannot be calculated. In 2014, Jetton et al. and Askenazi et al. introduced the neonatal modified KDIGO criteria (Table 14), which are based on the KDIGO diagnostic criteria [324, 325]. Similarly to the adult and pediatric KDIGO criteria, the neonatal modified KDIGO criteria define stages 1, 2, and 3 AKI primarily according to sCr values 1.5–1.9, 2.0–2.9, and ≥ 3 times higher than baseline, respectively (Table 15). Although the baseline sCr is the minimum value prior to AKI diagnosis, it is only established at age ≥ 3 months in Japan [317] and not in children under 3 months. The level of sCr in neonates immediately after birth is extremely close to the level of maternal sCr (generally ≤ 1 mg/dL) [326]. It peaks at days 0–3 and declines to a minimum value (0.2–0.5 mg/dL) over the following 1 week to 20 months [326–328]. (Note that prematurity (in terms of gestational age and birth weight) is reported to affect the postnatal sCr levels and the speed at which they decline [326, 328]). Going forward, it is necessary to collect data on Japanese neonates to establish their baseline sCr levels. It should be taken into consideration that the current absence of established baseline levels requires multiple measurements.

**Table 15:** Neonate modified KDIGO criteria

Stage	sCr criteria	UO criteria
Stage 0	No change or rise in SCr of < 0.3 mg/dL	UO ≥ 1 mL/kg/h
Stage 1	Increased in sCr of ≥ 0.3 mg/dL (48 h) or increase to 1.5–1.9 × baseline (7 days)	UO < 0.5 mL/kg/h × 6 h
Stage 2	Increase in sCr to 2.0–2.9 × baseline	UO < 0.5 mL/kg/h × 12 h
Stage 3	Increase in sCr > 3.0 × baseline or sCr ≥ 2.5 mg/dL or initiation of RRT	UO < 0.3 mL/kg/h × 24 h or anuria × 12 h

Reference sCr will be defined as the lowest previous value. sCr value of 2.5 mg/dL represents less than 10 mL/min/1.73 m^2^

*sCr* serum creatinine, *UO* urine output, *RRT* renal replacement therapy

Koralkar et al. used the neonatal modified KDIGO criteria to examine AKI and mortality in 229 very low-birth-weight infants both at 36 weeks of gestational age and with a birth weight of 500–1500 kg [329]; the very low-birth-weight infants diagnosed with AKI had a significantly higher mortality than those not diagnosed with AKI. In an examination of 455 very low-birth-weight infants using the neonatal modified KDIGO criteria, Carmody et al. found AKI to be associated with mortality and prolonged hospitalization [330]. In addition, a gestational age < 28 weeks was strongly associated with the onset of AKI; furthermore, all infants with a gestational age < 24 weeks were diagnosed with AKI, which indicates an association between prematurity and AKI. Rhone et al. used the neonatal modified KDIGO criteria to examine the association between the AKI onset and nephrotoxic medications (acyclovir, amphotericin B, gentamicin, ibuprofen, indomethacin, iohexol, tobramycin, and vancomycin) in 107 very low-birth-weight infants; consequently, these drugs were shown to be associated with the onset of AKI [331]. In an examination of 96 neonates with moderate to severe asphyxia who underwent therapeutic hypothermia, Sarkar et al. demonstrated that abnormal brain MRIs at 7–10 days of age were significantly more frequent in infants diagnosed with AKI according to the neonatal modified KDIGO criteria [332]. As detailed above, many recent studies have employed the neonatal modified KDIGO criteria for the diagnosis of neonatal AKI.

### Literature review

PubMed was searched for relevant studies published between January 1, 1980, and August 1, 2015, and papers related to the present CQ were identified from the search results.

## CQ9-2: Should biomarkers be used for the early diagnosis of AKI and for prediction of the survival outcomes in children?

*Recommendation*: The use of biomarkers for the early diagnosis of AKI or to predict the survival outcomes cannot be recommended in children.

*Strength of recommendation*: Not graded

*Quality of evidence*: C

### Summary of evidence

Many studies have indicated that biomarkers such as NGAL, cystatin C, L-FABP, IL-18, and KIM-1 may be useful for the early diagnosis of AKI and to predict the survival outcomes in children. However, interventions based on these indicators have not been reported to improve the renal or survival outcomes of AKI; therefore, their utility is limited.

### Commentary

Neutrophil gelatinase-associated lipocalin (NGAL) is a secretory protein that has a molecular weight of 25,000 Da and is secreted from activated neutrophils and tubular epithelial cells; the levels of NGAL in the blood and urine are known to be elevated in the hyperacute phase (i.e., the initial 2–4 h) of kidney injury. In an examination of 71 children undergoing a cardiopulmonary bypass (CPB) [333], the children who developed acute kidney injury (AKI) showed significantly elevated levels of serum and urinary NGAL 2 hours after the CPB, with areas under the receiver operating characteristic curve (AUC) of 0.998 and 0.906, respectively; this study was the first to indicate the utility of biomarkers for the early diagnosis of AKI. An examination of 311 children undergoing cardiac surgery for congenital heart disease registered at three institutions [334] also indicated that the urinary NGAL is useful for the early diagnosis of AKI, despite a relatively low AUC of 0.71. The urinary NGAL was also reported to be useful for the early diagnosis of AKI in a heterogeneous pediatric intensive care unit (PICU) patient cohort which had undergone mechanical ventilation and bladder catheterization [335]. Likewise, in a systematic review/meta-analysis of 19 studies [122], a subgroup analysis of 6 studies featuring populations of pediatric patients only demonstrated the utility of NGAL for the early diagnosis of AKI. With regard to the survival outcomes, two studies have reported that NGAL is significantly associated with mortality [336, 337].

Cystatin C is a low-weight molecular protein (molecular weight, approx. 13,000 Da) produced by nucleated cells all over the body. It is unaffected by environmental changes inside or outside the cells and is produced and secreted constantly; therefore, its concentration in the serum is constant. In addition, cystatin C is unaffected by factors such as inflammation, aging, the gender, the muscle mass, or exercise. The serum cystatin C passes freely through the glomerular basement membrane and is filtered by the glomerulus. As more than 99% of the serum cystatin C is reabsorbed by the proximal tubule and catabolized, healthy individuals excrete only a minimal amount of it in their urine. The serum cystatin C has been indicated to be useful for early, accurate diagnoses of AKI. The cystatin C concentrations in the serum and urine are known to increase 12–24 h after the onset of kidney injury. In an examination of 374 children undergoing CPB [338], AKI patients demonstrated significantly elevated serum cystatin C levels 12 and 24 h after the onset of AKI, with AUCs of 0.81 and 0.84, respectively; thus, the serum cystatin C was shown to be a useful biomarker for the early diagnosis of AKI. It was also reported to be useful for the early diagnosis of AKI in a study of 288 children undergoing cardiac surgery [339]. While measurement of the serum cystatin C is covered by insurance in Japan, that of the urinary cystatin C is not.

Interleukin-18 (IL-18) is an inflammatory cytokine induced in the proximal tubule. In a study of 55 children undergoing CPB [340], children with AKI demonstrated a significant acute phase (4–6 h after CPB) increase in their urinary IL-18 levels. The latter peaked at 12 h and remained high at 48 h. The AUC at 12 h (i.e., at the urinary IL-18 levels’ peak) was 0.75, demonstrating the utility of the urinary IL-18 as a biomarker for the early diagnosis of AKI. In a systematic review/meta-analysis of 18 studies [341], the urinary IL-18 was also shown to be useful for the early diagnosis of AKI in a subgroup analysis of five studies featuring populations of pediatric patients only. Measurement of the urinary IL-18 is not covered by insurance in Japan.

The L-type fatty acid-binding protein (L-FABP), kidney injury molecule-1 (KIM-1), and albumin are also known to show a marked increase in urine as a result of kidney injury; these biomarkers have been studied for their potential utility in the early diagnosis of AKI. The L-FABP is a protein with a molecular weight of 14,000 Da that is expressed in the liver, the small intestine, and the proximal tubular epithelial cells; since 2011, measurement of the L-FABP as a biomarker has been covered by insurance in Japan. In a study of 40 pediatric patients having undergone CPB [342], children who developed AKI demonstrated a significant acute phase (4 h after AKI onset) increase in the urinary L-FABP. KIM-1 is a membrane-spanning glycoprotein expressed in the proximal tubular epithelial cells. In a study of 40 children undergoing CPB [343], children with AKI demonstrated a significant acute phase (12 h after CPB) increase in KIM-1. Moreover, in a prospective study of 294 children undergoing cardiac surgery, the urinary albumin/creatinine ratios 0–6 h after surgery were useful for the prediction of AKI [344].

Due to the diversity of the pathologies involved in AKI and the decline in the glomerular filtration rate (GFR), the use of a single biomarker to increase the accuracy of early diagnosis is of limited efficacy. One attempt to increase the accuracy of biomarkers for the diagnosis of AKI is to assemble a “panel” that combines multiple biomarkers and the renal angina index (RAI), an indicator of the risk of AKI onset [345, 346]. Another advantage of panels is that, as each of the biomarkers that comprise them demonstrate favorable sensitivity and specificity at different periods, these different time phases may complement one another.

The uses of biomarkers have been studied in children, though not as often as in adults. Relevant studies indicate that these biomarkers may be useful for the early diagnosis of AKI and for the prediction of the survival outcomes. However, many of these studies involved relatively homogeneous populations, e.g., children undergoing CPB; the utility of these biomarkers has not been sufficiently assessed in populations of patients with diverse pathologies. Furthermore, the interventions based on these indicators have not yet been reported to improve the renal outcomes or survival outcomes of AKI; therefore, their utility is limited.

### Literature review

PubMed was searched for relevant studies published between January 1980 and July 2015, and papers related to the present CQ were identified from the search results.

## CQ9-3: Should fluid overload be considered as a blood purification indication for pediatric AKI?

*Recommendation*: When determining whether blood purification is indicated in pediatric AKI, we suggest considering the fluid overload assessment in addition to absolute indications.

*Strength of recommendation*: 2

*Quality of evidence*: C

### Summary of evidence

Many observational studies have reported that pediatric AKI non-survivors exhibit fluid overload compared to survivors. Few manuscripts have discussed fluid overload in neonatal AKI; there is little evidence to support prioritization of the fluid overload assessment for determination of the indication of blood purification in neonates.

### Commentary

In pediatric acute kidney injury (AKI), life-threatening conditions resistant to conservative therapy, such as electrolyte disorders (hyperkalemia, etc.), fluid overload (pulmonary edema, heart failure, etc.) metabolic acidosis, and uremia symptoms (pericarditis, impaired consciousness, convulsions, etc.) are absolute indications for blood purification just like in adult AKI; in these cases, blood purification must be initiated immediately. However, for relative indications that are not considered to be immediately life-threatening, the criteria for the initiation of blood purification have not yet been defined. No randomized controlled trials have assessed the indications for blood purification and the timing of its initiation in pediatric AKI.

Although they were only observational studies, many recent papers have reported that fluid overload at the initiation of blood purification affects the survival outcomes. Body water is known to account for a larger percentage of the body weight in children than in adults. The percent fluid overload (%FO) is considered to be useful for the assessment of fluid overload.

$$ {\displaystyle \begin{array}{l}\%\mathrm{Fluid}\ \mathrm{overload}\ \left(\%\mathrm{FO}\right)=\left(\mathrm{Fluid}\ \mathrm{in}\hbox{-} \mathrm{Fluid}\ \mathrm{out}\right)/\mathrm{PICU}\ \mathrm{admission}\ \mathrm{body}\ \mathrm{weight}\times 100\ \left(\%\right)\\ {}\mathrm{Fluid}\ \mathrm{in}-\mathrm{Fluid}\ \mathrm{out}:\mathrm{in}\hbox{-} \mathrm{out}\ \mathrm{balance}\ \mathrm{before}\ \mathrm{and}\ \mathrm{after}\ \mathrm{PICU}\ \mathrm{admission}\end{array}} $$

In 2001, Goldstein et al. conducted a single-center study [347], followed by a large-scale multicenter study of continuous renal replacement therapy (CRRT) for pediatric AKI whose results were reported in 2005 [348]. This study examined the predictors of survival and death in 116 children registered in the Prospective Pediatric Continuous Renal Replacement Therapy (ppCRRT) Registry who underwent CRRT for multiple organ failure. Even when controlling for the severity of the illness (as measured by pediatric risk of mortality [PRISM] score), the %FO at CRRT initiation was an independent predictor of survival; the %FO was significantly lower in survivors than in non-survivors (survivors 14.2 ± 15.9 vs non-survivors: 25.4 ± 32.9, *p* < 0.05), while the mortality was significantly higher when the %FO was > 20% (< 20%, 40% vs > 20%, 58%) at CRRT initiation. The same group later demonstrated that the %FO at the initiation of blood purification was correlated with mortality (< 10%, 29.4%; 10–20%, 43.1%; > 20%, 65.6%) [349]. Modem et al. reported that FO is a factor of poor survival outcomes [350]. Many studies of AKI in multiple organ failure [351–353], stem cell transplantation [354], and extracorporeal membrane oxygenation (ECMO) following cardiac surgery [355, 356] have also reported that a lower %FO at CRRT initiation is associated with more favorable survival outcomes. Similar results have also been reported in assessments of FO based on the body weight at hospital admission, at intensive care unit (ICU) admission, and at the initiation of blood purification [357]. Therefore, the early initiation of blood purification to prevent fluid overload may improve the survival outcomes; when determining whether blood purification is indicated in pediatric AKI, we suggest that the fluid overload assessment should be taken into consideration in addition to absolute indications.

However, all these results were obtained from observational studies; there is no high-quality evidence from interventional studies. In addition, a study of blood purification in children undergoing cardiac surgery failed to find an effective timing for initiation [358], while fluid overload was reported not to be an absolute predictor of the survival outcomes [359]. Unnecessary blood purification should be avoided in cases of mild AKI, in which the renal function recovers quickly. Blood purification carries serious complications, including catheter-related infection, an increased risk of bleeding from anticoagulation, and hemodynamic fluctuations unique to children of small constitution; therefore, the indication for blood purification and the timing of initiation must be considered comprehensively.

In neonatal AKI just like in pediatric AKI, renal replacement therapy (RRT) is considered when prolonged oliguria/anuria prevents the appropriate adjustment of the body fluid, electrolytes, and blood nitrogen level. The overall mortality in neonatal AKI is reported to range between 11.3 and 48.3% [360–370], while the mortality is reported to be 4.1–71.7% in premature neonates [329, 371–374], 13.9–70.0% in asphyxiated neonates [367, 375, 376], 2.9–11.6% in neonates undergoing a cardiopulmonary bypass/cardiac surgery [377–379], 71.2% in sepsis [380], and 50–100% in neonates with AKI who undergo blood purification [361, 365–367]. Due to the different definitions of AKI in these studies and to the major differences in the standards of neonatal intensive care medicine between countries and institutions, uniform comparisons of past studies are difficult. The risk factors for death in neonatal AKI include mechanical ventilation, hypervolemia (%FO ≥ 7%), chronic heart failure, a low birth weight, hypoxia, oliguria/anuria, dialysis, and metabolic acidosis [360]. The risk of death is particularly high in neonates with oliguria [360–364, 369, 375, 376]. However, no studies have discussed FO in neonatal AKI; there is little evidence to support prioritization of the fluid overload assessment when determining the indication of blood purification in neonates. Low-birth-weight infants present technical problems such as vascular access; however, the indication for acute blood purification in neonates must be determined comprehensively on a case-by-case basis.

### Literature review

PubMed was searched for relevant studies published between January 1980 and July 2015, and papers related to the present CQ were identified from the search results. Manuscripts that supplemented the commentary were hand searched as appropriate.

## CQ9-4: What modalities of blood purification therapy should be selected for pediatric AKI patients?

*Recommendation*: For pediatric AKI patients requiring blood purification, an appropriate modality tailored to the patient’s constitution and disease condition should be considered.

*Strength of recommendation*: Not graded

*Quality of evidence*: D

### Summary of evidence

Observational studies of children and neonates who underwent CRRT or other modalities of blood purification have been performed. However, there has been no evidence to demonstrate the effects of different blood purification modalities on the outcomes nor of their superiority to peritoneal dialysis.

### Commentary

The modalities of blood purification for acute kidney injury (AKI) include peritoneal dialysis (PD), extracorporeal intermittent renal replacement therapy (IRRT), and continuous renal replacement therapy (CRRT). In the past, PD was often the first choice; however, due to progress in the techniques of vascular access, in the types of catheters, the hemodialysis (HD) devices, and the pediatric intensive care management, extracorporeal CRRT has become more common. At present, the only studies that have compared PD and extracorporeal CRRT are observational studies [381, 382], and there is no evidence that one modality is superior to the other. However, as in adults, CRRT is considered preferable to IRRT for hemodynamically unstable patients. Many evidences that inform the blood purification modality selection for adults can be applied pediatric AKI; however, the incidence of pediatric AKI is < 1% in all hospitalized children [383] and 4.5% in children admitted to the ICU [384]. Differences in knowledge and health care resources between regions and institutions may greatly affect the selection of blood purification modalities. Further investigations are necessary to better inform the selection of suitable blood purification modalities.

In children (aside from neonates), blood purification can be performed safely with a combination of low priming volume and multipurpose blood purification devices. For vascular access, the size of the catheter is chosen to suit the patient’s constitution (Table 16). The standard values for the quantity of blood flow (QB), the dialysate flow rate (QD), and the filtration rate (QF) are 1–5 mL/kg/min, QB × 0.2–2.0, and 0–20% of the QB, respectively. With regard to circuit priming before the initiation of HD, a priming volume of ≥ 10% of the circulating blood volume causes hypotension at the initiation of dialysis; therefore, priming with blood products is preferred [385]. After priming with blood products, it is recommended dialyzing the priming blood in the circuit to adjust the electrolyte and acid-base balance and by removing the potassium and citric acid in the blood products.

**Table 16:** Blood purification for pediatric patients

Bodyweight (kg)		0~	1	2	3		5		10		15	20	25
Filter surface area (m^2^)				0.03					0.3		0.7		
Priming	Packed red blood cells ± albumin (A)	A	A	A	A	A	A	A/B	C	C	C	C	C
	Albumin (B)												
	Saline (C)												
Catheter size		18G	17G		6Fr		7Fr		8Fr		9Fr	10Fr	11Fr
QB	1~5 (mL/min) × bodyweight				3~15				10~50				
QD	QB × (0.2~2.0)				36~1800				120~6000				
QF	QB × (0~0.2)				0~180				0~600				
Anticoagulation	Nafamostat mesilate				1.5~3 mg/h				5~10 mg/h				
	Heparin				30~60 unit/h				100~200 unit/h				

*QB* quantity of blood flow, *QD* dialysate flow rate, *QF* filtration rate

In the past, PD used to be the method of choice for low-birth-weight infants (including neonates) due to the technical problems. However, extracorporeal blood purification can recently be performed safely. Extracorporeal blood purification has been technically possible in Japan since 2001, when blood purification devices (QB can be adjusted from 1 mL/min), filters, and other related devices became commercially available. In 2013, the Pediatric Acute Blood Purification Handbook described blood purification in neonates and the Guideline for Neonatal Extracorporeal Blood Purification was published in Japan. In addition, the Neonatal Extracorporeal Blood Purification Manual was published in 2014. Meanwhile, there have been many reports on blood purification primarily in pediatric patients (including some neonates) both in Japan and outside of Japan [348, 386, 387].

In blood purification for low birth-weight-infants (including neonates), vascular access is a specific and important issue; in addition to the standard central venous route, the umbilical arteries/veins and peripheral arteries can also be used (when the flow rate is low, the peripheral veins can sometimes also be used). There has been a Japanese case report of blood purification in an infant weighing < 500 g; however, blood purification in infants weighing < 2 kg is considered to require experienced skill. Central venous catheter size should be large. Although variations between institutions exist, the catheter sizes used for infants weighing 1, 2, and 3 kg are generally 17G, 15G, and 6Fr, respectively. As in pediatric patients, the circuit is basically primed with mixed blood in order to prevent hypotension. The blood preparation is recycled and dialyzed to remove potassium and citric acid before initiating the blood purification. Prevention for hypothermia is necessary. Details are described in the above-cited guidelines and handbooks (Table 16).

Outside of Japan, the blood purification of low-birth-weight infants (including neonates) was primarily consisted of peritoneal dialysis [388–390]. The improvements in blood purification devices have recently led to an increase in extracorporeal acute blood purification [391–393]. However, there have been no randomized controlled trials (RCTs) to examine the performance of extracorporeal blood purification in Japan or elsewhere.

The optimal blood purification modality also depends on the disease conditions. For AKI with acute brain injury, intracranial hypertension, or cerebral edema, CRRT (continuous hemodiafiltration [CHDF] or 24 h of PD) is recommended, as IRRT may cause intracranial hypertension, dialysis disequilibrium syndrome, and reduced blood pressure [394].

### Literature review

PubMed was searched for relevant studies published between January 1980 and July 2015, and papers related to the present CQ were identified from the search results. The literature needed for the commentary was manually extracted from PubMed as appropriate.

## CQ9-5: How should therapeutic strategies be discussed and determined in cases of neonates and children with AKI who have serious impairments and poor survival prognoses?

*Recommendation*: Medical care staff should first consider the patient’s present status and survival prognosis discuss the indication for renal replacement therapy among themselves. Afterwards, they should explain the advantages and disadvantages of the different treatments to the patient’s family and consult with them about suitable therapeutic strategies. Each patient should be dealt with as appropriate on a case-by-case basis and with reference to the “Guideline on Determining Medical Care of Children with Serious Diseases” of the Japan Pediatric Society.

*Strength of recommendation*: Not graded

*Quality of evidence*: D

### Summary of evidence

Despite the existence of multiple case reports and case series, there is no relevant high-level evidence.

### Commentary

Children with severe motor and intellectual disabilities caused by factors such as chromosomal abnormalities, multiple abnormality syndromes, and neonatal asphyxia (cerebral hypoxia) are estimated to occur in approximately 0.3 out of 1000 live births. Factors such as severe asphyxia and infection frequently cause acute kidney injury (AKI) in neonates. Neonates with AKI necessitating blood purification often present comorbid serious brain injury. Children with severe motor and intellectual disabilities that correspond to grades 1–4 of Oshima’s classification have a high risk of developing severe infections and frequently require renal replacement therapy (RRT) for AKI. However, most past reports of RRT in children with severe impairments, mostly from Japan, have involved the issues of chronic dialysis. Out of a total of 23 reports (37 patients), 20 of them (32 patients) described the initiation (or scheduled initiation) of peritoneal dialysis (PD), 2 (4 patients) described the initiation of chronic hemodialysis (HD), and 1 (1 patient) described PD and continuous hemodiafiltration (CHDF) for AKI. In another report, the patient was treated without initiating dialysis. Many of these reports have suggested that a multidisciplinary health care team should consider the patient’s case and should ultimately decide what to do after consulting the patient’s family. Meanwhile, reports from outside of Japan have stated that the frequency of peritonitis in PD for children with psychomotor retardation is the same as in children without psychomotor retardation if dedicated cooperation and support are provided. There have also been reports of dialysis initiation in children with chromosomal abnormalities [395–397]. These reports have demonstrated that RRT can be performed relatively safely in children with severe motor and intellectual disabilities. However, the medical staff experience a great physical and psychological burden; therefore, the health care team also needs support.

There are no definitive criteria upon which to determine the indication for RRT in children with severe impairments; thus, it must be considered on a case-by-case basis. The health care team should decide on a therapeutic strategy after considering the patient’s present status and long-term survival prognosis among themselves, explaining the nature of the treatments to the patient’s family, and presenting the respective advantages and disadvantages of treatment versus no treatment. Various guidelines [30–32] can be referred as well. This concept is called shared decision-making; essentially, health care professionals must share information with the patient’s family and decide on a therapeutic strategy together. The process is described below.

#### Therapeutic strategy discussion among the health care team

Before providing information to the patient’s family, the health care staff must gather information and share it among themselves in order to determine the patient’s present status. Discussions should not only include the attending physician’s department, but also intensive care specialists, neonatal intensive care specialists, and nurses; when necessary, clinical psychologists, a palliative care team, medical social workers, and other departments and disciplines should also be included. Based on these discussions, conceivable treatments should be identified as options, and the problems and invasiveness of each option should be abstracted (for example, for acute blood purification, these include complications associated with catheter insertion, the risk of hypotension associated with dialysis initiation, blood transfusion). Suitable strategies are then examined based on a prediction of the patient’s prognosis (survival prognosis and sequelae) and on the consideration of the advantages and disadvantages of the potential treatments.

When considering withholding or discontinuing treatment, the relevant facility’s institutional review board may be convened, or a conference may be held to discuss ethical issues.

#### Explanation to the patient’s family

When explaining therapeutic strategies to the patient’s family, the parents must always be present; other individuals may attend the explanation if requested by the parents (grandparents, etc.). The name of the child’s illness, its disease condition, the respective advantages and disadvantages of treatments such as blood purification (including their complications) versus no treatment, and the prognosis (sequelae and survival prognosis) should be explained comprehensively in a way that is easy to understand. Important information should be provided in writing. Moreover, even when acute blood purification is to be performed, it must be explained that permanent RRT may be necessary, thereby placing a burden on the patient and their family (which also requires explanation). In addition, the family must be informed that even after a strategy is decided, it can be reconsidered if they change their minds.

The content of this explanation, the way it is explained, and the course by which a strategy is chosen must be written in the patient’s medical record. In particular, when treatment is withheld, it is important to record the course and content of the discussion that led to the treatment withdrawal. When the patient’s family and the health care team cannot agree on a strategy, advice should be sought from a committee comprising the institutional review board and many other experts.

#### Subsequent follow-up and reconsideration of the treatment strategy

Even after a strategy is decided, the patient’s family will require continuous mental support. After blood purification is initiated, the patient’s impairment may progress irreversibly, thereby requiring discontinuation of the treatment. On the other hand, even if the patient’s family initially decides not to perform the treatment, the treatment may later be performed if they change their minds (or for other reasons). These reconsiderations of the therapeutic strategies require a new round of discussion. Moreover, when changing the therapeutic strategy, a consensus must be obtained among the health care team as appropriate.

If the patient’s family wishes to discontinue dialysis, it is necessary to confirm that this is not based on temporary emotion but on a careful consideration and sufficient understanding of the child’s status. The patient may also die shortly after discontinuing treatment; therefore, when deciding to do so, the timing of the discontinuation must also be discussed.

The above procedure is also followed for comorbid severe brain injury associated with acquired causes, such as acute encephalitis/encephalopathy and head trauma. When the patient him/herself is evidently conscious and is capable of expressing their will but has a poor survival prognosis (such as in the case of older children with terminal malignancy), the patient’s own will must be respected and prioritized above all else. In such cases, the question of how much medical information to convey to the patient must first be discussed with the patient’s family and agreed in advance.

### Literature review

PubMed and Ichushi-Web (Japanese language) were searched for relevant studies published up to August 30, 2015, and papers related to the present CQ were identified from the search results. References in Japanese are not shown in this article.

### Chapter 10: AKI in the elderly and ethical aspects

#### Aging as a risk factor for AKI occurrence

As Japan has become a super-aged society, it is increasingly crucial to understand the pathologies in which age is a risk factor and to take preemptive measures to prevent these pathologies in order to tackle diseases with no established treatments. A typical example of these pathologies is acute kidney injury (AKI). Elderly people account for a large and constantly increasing percentage of AKI patients [398]. In addition, many observational studies published in the last 25 years have found aging to be a significant risk factor for AKI onset [34, 399].

Pre-AKI renal impairment is a risk factor for AKI; chronic kidney disease (CKD) patients are at a high risk of developing AKI. With the base of hypertensive nephrosclerosis, aging is associated with a reduced glomerular filtration rate (GFR); therefore, aging is conceivably an underlying factor of CKD, which in turn can be considered a universal risk factor for AKI [400, 401]. In the present guideline’s examination of the risk factors for individual AKI, age was considered to be an independent risk factor for the onset of AKI in cardiac surgery (“CQ3-1: What should be assessed as risk factors for AKI development in cardiac surgery?” section), acute heart failure (“CQ3-3: What should be assessed as risk factors for AKI development in heart failure?” section), and sepsis (“CQ3-4: What should be assessed as risk factors for AKI development in sepsis?” section). Although it is not covered by any CQ in the present guideline, dehydration-induced pre-renal AKI, which is an important aspect of community-acquired AKI, restricts the renal blood flow in elderly people due to their already low fluid volume and to arteriosclerosis; therefore, elderly people are at a high risk of developing dehydration-induced AKI [402]. Drugs such as renin-angiotensin-aldosterone system inhibitors, diuretics, non-steroidal anti-inflammatory drugs (NSAIDs), and vitamin D preparations (which cause hypercalcemia)—the latter two of which are often used by elderly people—are involved in the increase in AKI among the elderly [403]. Elderly people are also known to be at a high risk of drug-induced AKI (contrast agents, aminoglycosides, etc.) [404, 405]. Therefore, in order to prevent AKI, elderly people’s exposure to these drugs must be minimized. Urinary tract obstructive kidney injury and ANCA-associated, vasculitis-induced rapidly progressive glomerulonephritis (RPGN) are also common causes of AKI in the elderly.

The present guideline recommends the use of the KDIGO criteria for the diagnosis of AKI; however, caution is necessary in applying these criteria to elderly patients. Although the KDIGO diagnostic criteria for AKI depend on the baseline renal function, it is often unknown in clinical settings; therefore, it is permissible to use the serum creatinine (sCr) as back-calculated from the MDRD formula by assuming an estimated glomerular filtration rate (eGFR) of 75 (or from the sCr-eGFR predictive equation for Japanese people). However, a standard eGFR of 75 often overestimates the renal function in elderly people; consequently, the sCr as back-calculated from eGFR formulas is underestimated, which causes an increase in false positives in the AKI diagnosis (overdiagnosis). Due to their diminished capacity for renal recovery, AKI easily progresses to a severe state in elderly people. Because of the physical frailty and of cardiovascular complications, it is highly likely that the survival and renal outcomes predicted for adults do not apply to elderly people. Based on the above, elderly people, who are at a high risk of developing AKI, require highly accurate AKI diagnoses; therefore, the development of dedicated AKI diagnostic criteria for elderly people may be needed.

#### Blood purification in elderly AKI patients

Aging is an evident high-risk factor for AKI development [34, 399]. In Japan and other developed nations, the incidence of AKI has been increasing as the population ages; this trend is particularly pronounced in men [398, 406, 407]. Blood purification is more often required in elderly patients (particularly those aged over 75) [408]. In a Turkish observational study, blood purification was performed in 43 of 193 patients (22%) with a mean age of 79.99 years who were diagnosed with AKI as defined by the KDIGO classification; when including the 16 patients (12.7%) who required blood purification after discharge, a total of 37.7% of the patients required blood purification [409].

In AKI patients—including elderly ones—whose AKI progresses to an advanced stage and presents uremic symptoms, blood purification undoubtedly improves the survival outcomes [410]. However, there have been no randomized controlled trials (RCTs) that enrolled elderly patients with advanced AKI with the survival outcome as the primary endpoint nor have there been any relevant systematic reviews. However, there has been a retrospective cohort study of elderly AKI patients. Liu et al. examined the factors that affected the survival outcomes of 41 elderly AKI patients aged 80–100 years who required continuous renal replacement therapy (CRRT) in Beijing, China [411]. In the AKI patients who underwent CRRT, the APACHE II score was the factor most strongly associated with the survival outcomes; the number of involved organs and hypoalbuminemia was also indicated to be important, while the age itself was unrelated to the survival outcomes. These results are not limited to elderly patients, but are relatively common to all AKI patients; they may indicate that if AKI has reached an advanced stage, blood purification should be considered even in elderly patients. However, in a similar study by Kayatas et al. involving patients within a slightly broader age range (≥ 65 years), a reduced blood pressure, high CRP levels, and low hemoglobin (Hb), aging was also found to be associated with the AKI outcomes [409]. Moreover, several studies have noted racial differences in the outcomes; among elderly ICU patients who required blood purification, the outcomes were worse for non-Caucasian patients [412, 413].

In elderly AKI patients, the outcomes are often affected not only by AKI but also by existing comorbidities. This is also observed in maintenance hemodialysis (HD) patients and elderly people in general [414]. Elderly AKI patients in ICUs are reported to commonly exhibit evident dementia and symptoms of delirium [412]. In addition, the AKI morbidity is high in frail elderly people; the latter is highly likely to require blood purification, and their activities of daily living are reported to decline progressively [ [413, 415]]. Therefore, when considering whether to perform blood purification in an elderly AKI patient, the chronological age alone is not sufficient; the severity of the AKI, the speed of its progression, and details of the patient’s pre-AKI health status may also be necessary. The above has also been stated in a limited number of studies. However, in elderly AKI patients who did not demonstrate any major health problems before developing AKI, we do not recommend the needless avoidance of blood purification simply because of age. Conversely, AKI patients with multiple comorbidities and low activities of daily living before developing AKI are highly likely to have a poor renal and survival prognosis, which makes it necessary to consider whether blood purification should be performed or not [415]. Giving a definitive answer to this question would require prospective RCTs involving large numbers of elderly AKI patients, as well as sub-analyses to determine the effective groups. Coca at Yale University recommends that such RCTs involving elderly AKI patients should be conducted [416].

On the patient’s side, medical economic factors would normally be the greatest determinants of whether to undergo blood purification; however, since Japan has abundant public health insurance, medical economic aspects (burdens of expenses) do not have a major impact on patients’ decisions. Therefore, the decision whether to undergo blood purification is considered with social factors on the patient’s side, medical perspectives, and the medical institution’s treatment capacity on the medical side.

#### Progression from AKI to CKD in elderly patients

The renal outcomes of AKI are not favorable. Observational studies have shown that 20–50% of AKI survivors progress to CKD. AKI is not only involved in the de novo development of CKD, but it may also accelerate existing CKD. When AKI develops in a person with a previously normal renal function, if the renal function does not recover to pre-AKI status, one of the following three pathways will unfold: (1) progression to end-stage kidney disease (ESKD) after the onset of AKI (AKI to ESKD), (2) incomplete recovery of the renal function from AKI and progression to CKD (AKI to CKD), and (3) temporary recovery of the renal function from AKI, but subsequent progression to CKD (AKI to subclinical CKD). Furthermore, it has been shown that 30% of AKI patients have underlying CKD. This represents a fourth pathway: AKI to worsening CKD.

The prevalence of CKD in adults is estimated to be ≥ 10%. As the renal function declines with age, the prevalence of CKD is higher in the elderly; CKD affects 30–40% of people aged ≥ 65 years. Aging has been identified as a risk factor for post-AKI progression to CKD, along with diabetes, hypertension, heart failure, renal impairment, and hypoalbuminemia. The differences in the AKI incidence and the renal function outcomes of elderly and non-elderly people have not been examined in sufficient detail. However, in light of the high prevalence of CKD in the elderly and of the involvement of aging itself in the risk of progression from AKI to CKD, it is rather unlikely that the post-AKI renal function outcomes would be more favorable in elderly people than in non-elderly people. It is necessary to pay attention to the prevention and early detection of AKI, and its progression to a severe state.

The renal outcomes of AKI in elderly people were analyzed in a study of people enrolled in Medicare, the American health insurance system for the elderly (aged ≥ 65 years) [417]. Of the more than 230,000 people examined, CKD was present in 12%, while AKI had developed in 3.1%. Of the people who had developed AKI, 34% had prior CKD (AKI + CKD). The post-AKI survival rates of AKI + CKD patients were worse than those of patients with AKI alone. This study also analyzed the risks of progression to ESKD within 2 years of discharge; the hazard ratios for the development of ESKD in AKI + CKD, AKI only, and CKD only were 41.19, 13.0, and 8.43, respectively. These results indicated that elderly people with CKD who develop subsequent AKI experience poor renal outcomes.

The severity and frequency of AKI have also been reported to be independently involved in the risk of progression to CKD. One study retrospectively analyzed the relationship between the post-hospitalization development of AKI and the prognosis in a cohort of elderly people hospitalized for myocardial infarction who were Medicare beneficiaries [418]. In the analysis—in which the patients were divided into four quartiles based on sCr increases of 0.1–3.0 mg/dL—the quartile of patients with the largest percentage increase in sCr demonstrated high rates of pre-existing diabetes, hypertension, myocardial infarction, congestive heart failure, and cerebrovascular injury, as well as a reduced renal function. After adjusting for these factors, the percentage increase in the sCr demonstrated significant correlations with the percentage of post-AKI progression to end-stage renal failure and death. In elderly patients, the AKI severity was associated with the renal and survival outcomes. An association was also observed between the number of AKI episodes and the rate of progression to CKD. In a study of American veterans with comorbid diabetes, patients with multiple AKI episodes demonstrated a higher rate of progression to stage G4 CKD than those with a single AKI episode [419]. Given that a high percentage of elderly people have CKD and that the post-AKI renal function outcomes are poor in patients with comorbid CKD, practitioners should be cautious about the development of AKI in non-elderly and older people.

#### Ethical considerations relevant to AKI treatment in elderly people

Elderly people are at a high risk of AKI and have poorer renal and survival outcomes than young people. This point has already been discussed in the present guideline, and it has also been covered in many studies [415, 417, 420]. In a study comparing dialyzed AKI patients with not-yet-dialyzed AKI patients, Wilson et al. concluded that dialysis causes more harm than no dialysis when the sCr is below 3.8 mg/dL; the authors’ opinion was that dialysis is harmful in the presence of a decreased muscle mass, i.e., in frail patients [413]. The message of these findings is that in elderly patients, AKI must be recognized not as a transient disease condition that can be cured but rather as a serious status that leads to prolonged hospitalization, more complications, and a higher risk of death. Moreover, the decision to initiate dialysis in elderly AKI patients must be understood not only in terms of its effects on the prognosis (i.e., the survival prognosis, progression to chronic dialysis) but also of its major effects on the patient’s quality of life (QOL).

##### Points of note in relation to elderly AKI patients’ treatment

Crews et al. demonstrated that in elderly AKI patients, earlier dialysis initiation may in fact cause harm. The implementation of shared decision-making (as described below) for dialysis initiation enables more patient-centered care, and the elderly who participate in this process tend to forgo dialysis initiation [421]. When deciding whether to initiate dialysis in an elderly AKI patient, it is important to make a comprehensive determination of the indication based on more than the disease condition and to obtain consent from the patient (or their guardian) by dialoguing with them. This process, which is called “shared decision-making,” is described in a guideline published by the Renal Physicians Association in the United States entitled “Shared Decision Making in the Appropriate Initiation of and Withdrawal from Dialysis, 2nd Edition.” We present here a proposed “dialysis assessment form for elderly AKI patients,” which is based on a modification of the above guideline. Another potentially helpful resource is a process notebook that was developed in Japan to consider the initiation of dialysis in elderly patients with the patient’s cooperation. Going forward, we hope that this perspective will lend further momentum to shared decision-making in the consideration of elderly AKI patients’ treatment.

The aging of society and the consequent increase in social welfare expenses

The Japanese society has been aging at a rate that is unparalleled in the world, and this trend is predicted to continue. In the 2014 fiscal year (FY), the aging rate in Japan reached 26.0%; specifically, individuals aged 65–74 years accounted for 13.4% of the overall population, while those aged ≥ 75 years represented 12.5% of the population. The aging rate is predicted to exceed 30% by 2025. In FY 2012, Japan’s total social welfare expenditure reached \108.5568 trillion (~ $977 billion), its highest level ever. The percentage of national income spent on social welfare expenses has risen from 5.8% in 1970 to 30.9%, and the benefits paid to the elderly accounted for 68.3% of these expenses in FY 2012. Of the approximately \45 trillion (~ $405 billion) spent on national health care in FY 2012, approximately \18 trillion (~ $162 billion; 44%) was spent on late-stage elderly people (age ≥ 75 years, 12.5% of the overall population). It must be recognized that these medical economic factors may also have a considerable influence on the treatment of AKI in elderly patients.
